# Forecasts of mortality and economic losses from poor water and sanitation in sub-Saharan Africa

**DOI:** 10.1371/journal.pone.0227611

**Published:** 2020-03-20

**Authors:** David Fuente, Maura Allaire, Marc Jeuland, Dale Whittington

**Affiliations:** 1 School of Earth, Ocean & Environment, University of South Carolina, Columbia, South Carolina, United States of America; 2 Department of Urban Planning & Public Policy, University of California, Irvine, California, United States of America; 3 Sanford School of Public Policy and Duke Global Health Institute, Duke University, Durham, North Carolina, United States of America; 4 Departments of Environmental Sciences & Engineering and City & Regional Planning, University of North Carolina at Chapel Hill, Chapel Hill, North Carolina, United States of America; 5 Global Development Institute, University of Manchester, Manchester, England, United Kingdom; Helen Keller International, SIERRA LEONE

## Abstract

This paper presents country-level estimates of water, sanitation and hygiene (WASH)-related mortality and the economic losses associated with poor access to water and sanitation infrastructure in sub-Saharan Africa (SSA) from 1990 to 2050. We examine the extent to which the changes that accompany economic growth will “solve” water and sanitation problems in SSA and, if so, how long it will take. Our simulations suggest that WASH-related mortality will continue to differ markedly across countries in sub-Saharan Africa. In many countries, expected economic growth alone will not be sufficient to eliminate WASH-related mortality or eliminate the economic losses associated with poor access to water and sanitation infrastructure by 2050. In other countries, WASH-related mortality will sharply decline, although the economic losses associated with the time spent collecting water are forecast to persist. Overall, our findings suggest that in a subset of countries in sub-Saharan Africa (e.g., Angola, Niger, Sierra Leone, Chad and several others), WASH-related investments will remain a priority for decades and require a long-term, sustained effort from both the international community and national governments.

## 1. Introduction

The magnitude of water and sanitation problems in developing countries is commonly described using two indicators: 1) coverage with ‘improved’ services; and 2) the number of deaths (or episodes of illness) due to water and sanitation-related diseases [1, 2, 3, 4]. These two indicators are obviously related: poor coverage leads to increased deaths and episodes of illness. Data on these two indicators are typically presented for current (‘status quo’) conditions. Sometimes analysts make comparisons between past coverage statistics and current conditions in order to show the rate of progress a country is making. Only rarely do analysts look ahead and attempt to forecast how these two indicators appear likely to change in the future, and to understand the economic consequences of these trends.

Previous research has shown that deaths and episodes of illness due to poor water and sanitation conditions are falling rapidly in most low- and middle-income countries and seem likely to continue to decline over the next few decades [5]. By 2050 WASH-related deaths will largely disappear in many parts of the developing world as economic growth and the demographic transition combine to expand coverage with improved water and sanitation services, and as better nutrition, health services, and housing all contribute to enhanced human well-being. On a regional basis, the primary exception to this good news is sub-Saharan Africa, one of only two regions that did not achieve the Millennium Development Goals (MDGs) of halving the population without access to improved water sources and sanitation facilities by 2015 [1]. Based on demographic and economic growth projections, [5] suggested that without targeted interventions, deaths due to WASH-related diseases and the economic losses associated with poor access to water and sanitation services will remain at high levels in many countries in sub-Saharan Africa.

At first glance this grim forecast for sub-Saharan Africa is surprising because some African countries have experienced strong increases in economic growth over the past few decades [6]. Moreover, throughout the world (including in Africa), child mortality rates are declining and this decline is accelerating [7]. In this paper, we examine forecasts for WASH-related outcomes for sub-Saharan Africa in greater detail than in previous work, looking carefully at the underlying dynamics behind the high mortality from WASH-related diseases in Africa, as well as the economic losses associated with poor access to water and sanitation services. We show that the regional averages for sub-Saharan Africa obscure important differences in WASH-related outcomes across countries.

Specifically, in this paper recent country-level data and forecasts are used to identify four groups of countries with different WASH-related mortality rate trajectories. We then examine the economic losses associated with WASH-related mortality and morbidity as well as time spent collecting water. Countries in the first group currently have high WASH-related mortality rates, and our projections indicate that these rates will not decline much by 2050 if current trends continue. Countries in the second group have moderate WASH-related mortality rates today compared with the rest of sub-Saharan Africa, but these remain above current rates in South Asia, the other region of the world with countries with significant WASH mortality, by 2050. Countries in the third group start with moderate mortality rates, and these rates decline to negligible levels before 2050, as do those in South Asia. Countries in the fourth group start with low WASH-related mortality and these rates remain low throughout the simulation period.

The projected trajectories of the sub-Saharan African countries in the third and fourth groups look much like those of the rest of the world [5]. For these countries, health-related economic losses and high mortality from poor water and sanitation conditions will soon be a thing of the past. Economic losses in these countries are forecast to remain low and consist primarily of losses associated with the time burden of water collection. However, countries in the first and second groups need special attention from the international community. Unfortunately, over our projection period to 2050, the majority of the population of sub-Saharan Africa will reside in countries that are not making sufficient progress to reduce WASH-related mortality and the economic losses associated with poor water and sanitation conditions.

In the next, second section of the paper we discuss the current, status quo conditions for countries in sub-Saharan Africa with respect to the variables of special interest for our analysis, including population, gross domestic product (GDP) per capita, urbanisation, water and sanitation coverage, WASH-related mortality, and time households spend collecting water. Our forecasts begin from these baseline conditions. The third section summarises the modelling framework, highlighting the differences from previous forecasts of global trends in WASH-related mortality and economic losses. The fourth section presents the results, including sensitivity analyses. In the fifth and final section we offer concluding observations.

## 2. Background

### 2.1 Water infrastructure coverage in sub-Saharan Africa

Water and sanitation infrastructure coverage in sub-Saharan Africa lags behind other regions of the world; our forecasts of future coverage thus start from a low baseline. The World Health Organization’s and UNICEF’s Joint Monitoring Programme (JMP) offers the most up-to-date, internally consistent set of indicators of the percentage of a country’s population that has access to improved water and sanitation conditions.

The JMP publishes coverage statistics for two definitions of improved water services. The first is the simplest and most straightforward: “a piped water connection on the premises”. This definition includes both yard taps (outdoor plumbing only) and piped water delivered inside the house (indoor plumbing). The second is “an improved water source that by the nature of its construction, adequately protects the source from outside contamination in particular with fecal matter” [1]. The JMP classifies all of the following as improved sources: 1) piped into dwelling, plot, or yard; 2) public tap/standpipe; 3) tube well/borehole; 4) protected dug well; 5) protected spring; and 6) rainwater collection. “Piped into the dwelling, plot, or yard” (item no. 1) is one of the six types of improved sources, so the first definition is a subset of the second definition, i.e. reported coverage using the second definition will always be higher than reported coverage using the first definition.

**Fig 1:**
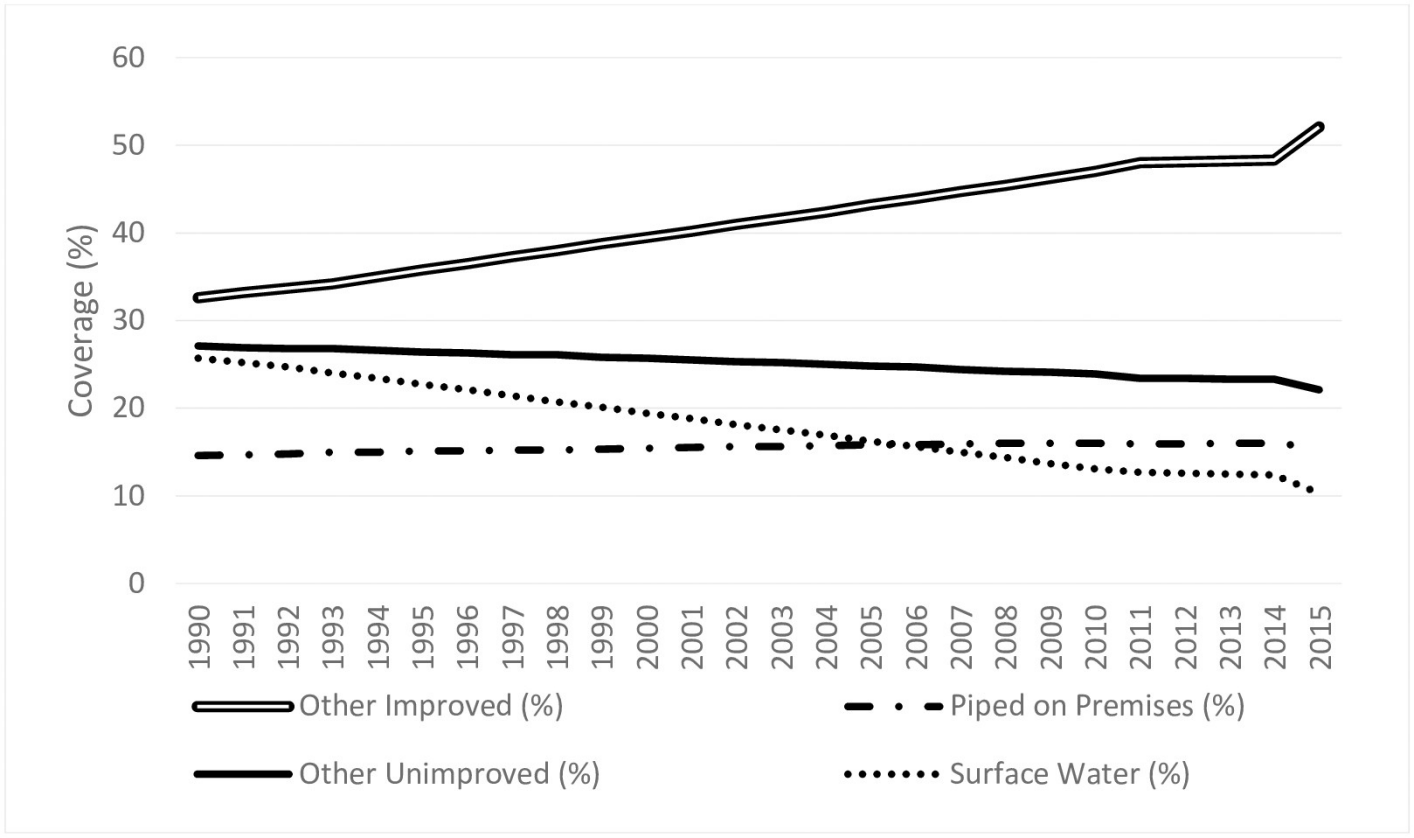
Trends in access to drinking water sources in sub-Saharan Africa (1990–2015). Source: [1].

Fig 1 shows the changes in coverage in sub-Saharan Africa from 1990 to 2015 for four categories of water sources: 1) piped water on premises (improved); 2) other improved; 3) other unimproved; and 4) surface water source (unimproved). Coverage with ‘other improved sources’ grew substantially over the period, from 34 percent to over 50 percent. ‘Piped water on premises’ actually decreased over the 25-year period as a percentage of the population, although the total number of people with piped water increased because the population grew. The big drop in the percent of the population using unimproved sources occurred for ‘surface water’, which fell from 24 percent to approximately 10 percent. This drop was in part attributable to increasing urbanization over this period and households transitioning from surface water to ‘other improved sources’.

### 2.2 WASH-related mortality rates in sub-Saharan Africa

No one really knows how many people die each year due to illnesses caused by poor water and sanitation conditions. Reports that 2 million children die annually due to WASH-related diseases are estimates, not precise counts. Many deaths in developing countries are not recorded in official statistics, and when deaths are recorded, the cause of death is often unknown. Global estimates of deaths due to WASH-related diseases are calculated using comparative risk methods [8]. These methods use data on what is known about baseline water and sanitation conditions and the health risks of such conditions to infer likely episodes of illness and deaths. Data on baseline conditions, the causal relationship between conditions and episodes of illness, and the relationship between episodes and deaths (case fatality rates) are thus subject to significant uncertainty [9]. As a result, there is also considerable uncertainty surrounding the final estimates of mortality due to poor WASH conditions.

[10, 11] provide estimates of diarrhoea incidence in countries in sub-Saharan Africa and other regions of the world; the [12] has estimated WASH-related mortality data (S1 Appendix). The estimates of diarrhoea incidence in sub-Saharan Africa are nearly 30 percent higher than in South Asia (based on diarrhoea incidence data from [10] and population data from [13]. Yet, WASH-related mortality rates were estimated to be twice as high in 2004 in sub-Saharan Africa as in South Asia [12].

Despite these unresolved questions about the underlying determinants of the sub-Saharan WASH-related mortality estimates (see S2 Appendix), for the purposes of our analysis, we use the WHO mortality estimates in our forecasting model. However, just as with the improved water coverage estimates, we emphasize that there remains considerable uncertainty about their accuracy.

## 3. Modelling strategy and data

**Fig 2:**
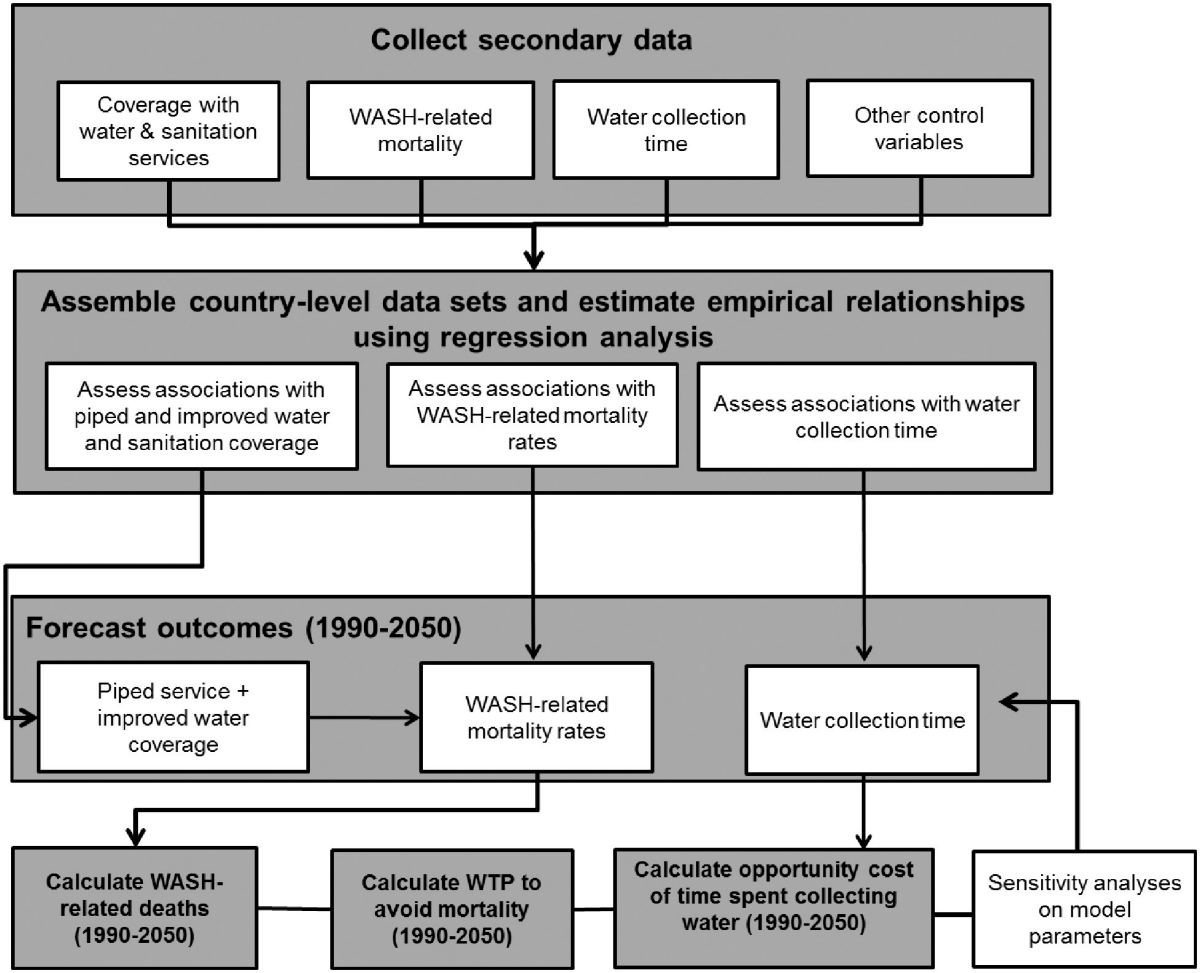
Schematic diagram of the simulation model (Source: Adapted from [5]).

In this paper, we use the simulation model developed and discussed in [5] to forecast the WASH mortality rate and annual WASH-related deaths from 1990 to 2050. The simulation model consists of three primary steps. In the first step we use regression analysis and global, country-level data to estimate: 1) the association between GDP, urbanisation, and access to improved water sources; 2) the association between GDP, access to improved water sources, and WASH-related mortality; and 3) the association between GDP, access to improved water sources, urbanisation, and the time households spend collecting water. In the second step, we combine the parameter estimates obtained in these three regressions with exogenous projections of GDP, population, and urbanisation (S3 Appendix) to forecast access to improved water sources, WASH-related mortality, and time households spend collecting water through 2050 for each country. In the third and final step, we estimate the economic losses associated with the forecast health and time-related outcomes. A schematic of the forecasting model is shown in Fig 2.

In the first step, we estimate the association between GDP and access to improved water sources (Eq 1).

$$Coverage_{it}=\kappa_{0}+\kappa_{Y}\cdot \text{ln}\left(Y_{i,t-1}\right)+\kappa_{2}\cdot Z_{ij,t}+\delta_{i,t}+\mu_{i}$$ (1)

where *Coverage*_*it*_ is access to improved water sources, Y_i,t-1_ is lagged income, Z_ij,t_ is a vector of *J* control variables (WHO regional dummy variables, linear time trend, year dummy variables, percentage urban population, income inequality, several governance variables, bilateral aid received for WASH, and a set of time–region interaction variables) measured at time *t* for country *i*, δ_i,t_ is a time-varying error term, *μ*_*i*_ is a time-invariant error term, and *κ*_*n*_ are the model coefficients estimated using regression analysis. This regression equation is estimated for both JMP definitions of improved water coverage. ([5] provides additional details about our estimation strategy and regression results. Sub-Saharan Africa is the omitted region in the global regression).

We then estimate the relationship between WASH-related mortality ($d_{it}^{WASH}$), access to improved water sources (*W*_*ilt*_), and other control variables (Eq 2).

$$d_{it}^{WASH}=\alpha_{0}+\alpha_{Y}\cdot \text{ln}\left(Y_{it}\right)+\beta \cdot W_{ilt}+\gamma \cdot X_{ikt}+\delta_{i,t}+\varepsilon_{i}$$ (2)

where *d*_*it*_^*WASH*^ is the WASH-related mortality in country *i* at time *t*, *Y*_*it*_ is per capita GDP (1990 international dollars), *W*_*ilt*_ is a vector of *l* water coverage variables, and *X*_*ikt*_ is a vector of *k* other control variables (regional dummy variables, percentage urban population, income inequality, fertility, literacy, and several governance variables: democracy-autocracy score (polity), regime durability, and an indicator for coups d’état). Eq 2 is estimated using both indicators of coverage—piped water on premises and access to an improved water source—as independent variables associated with WASH-related mortality.

Finally, we estimate the relationship between the time households spend collecting water using Eq 3.

$$t_{it}^{collection}=\theta_{0}+\theta_{Y}\cdot \text{ln}\left(Y_{it}\right)+\tau \cdot W_{ilt}+\gamma \cdot X_{ikt}+\delta_{i,t}+\varepsilon_{i}$$ (3)

where, $t_{it}^{collection}$ is the average one-way water collection time for households in country *i* at time *t*, *Y*_*it*_ and *W*_*ilt*_ are defined as in Eq 2, *X*_*ikt*_ is a vector of control variables (per capita GDP, urbanisation, income inequality, regime durability, democracy-autocracy score, and indicator variables for the source of the water collection time data i.e. MICS, DHS, and WHS). (Model specification and regression results are presented in [14]).

The relationships we estimate in Eqs 1, 2 and 3 should not be interpreted as implying a causal link between GDP per capita, access to improved water sources, decreased mortality or the time households spend collecting water. However, the associations between these variables are strong, which increases our confidence that these associations can be used for our forecasting purposes [5].

To forecast access to improved water sources over the simulation period, the parameter estimates obtained in Eq 1 are combined with exogenous projections of GDP and urbanisation, as shown in Eq 4.

$$Coverage_{it}=Coverage_{it-1}+\kappa_{Y}\cdot \Delta Y_{it}+\kappa_{urban}\cdot \Delta Urban_{it}$$ (4)

where *Coverage*_*it*_ is the coverage level in country *i* at time *t*, *Coverage*_*it-1*_ is the coverage the previous year, *κ*_*Y*_ is the elasticity of coverage with respect to GDP from Eq 1, Δ*Y*_*it*_ is the change in GDP from time *t-1* to time *t*, *κ*_*urban*_ is the elasticity of coverage with respect to urbanisation from Eq 1, and Δ*Urban*_*it*_ is the change in the percentage of the population living in an urban area from time *t-1* to time *t*. Eq 4 is used to forecast coverage levels for both the JMP’s least restrictive definition of improved water as well as piped water on premises using the coverage level specific elasticities estimated from Eq 1.

After forecasting coverage with improved and piped water services, the simulation model then estimates the WASH mortality rate in each country as described in Eq 5.

$$d_{it}^{WASH}=d_{it-1}^{WASH}+\alpha_{Y}\cdot {\Delta \text{Y}}_{it}+\beta_{imp}\cdot \Delta Improved_{it}+\beta_{piped}\cdot \Delta Piped_{it}+\gamma_{temp}\cdot \Delta T_{it}$$ (5)

Where $d_{it}^{WASH}$ is the WASH mortality rate defined as in Eq 2; *α*_*Y*_, *ϐ*_*imp*_, and *ϐ*_*piped*_ are the elasticities of WASH mortality with respect to GDP, access to improved water, and access to piped water from Eq 2, respectively; *γ*_*temp*_ is the elasticity of WASH mortality with respect to temperature; *ΔY*_*it*_ is the change in GDP from period *t-1* to *t*; Δ*Improved*_*it*_ is the change in access to improved water; Δ*Piped*_*it*_ is the change in access to piped water; and Δ*T*_*it*_ is the change in temperature. We then calculate the annual WASH-related deaths in each country by multiplying the projected WASH mortality rate in each period by the projected population for each country during that period.

Eq 6 describes how the simulation model forecasts water collection times.

$$t_{it}^{collection}=t_{it-1}^{collection}+\theta_{Y}\cdot \Delta Y_{it}+\gamma_{urban}\cdot \Delta Urban_{it}$$ (6)

where, $t_{i,t}^{collection}$ is the average one-way water collection time for households in country *i* at time *t*; *ΔY*_*it*_ is the change in GDP from period *t-1* to *t*; Δ*Urban*_*it*_ is the change in the percentage of population that lives in an urban area; and *θ*_*Y*_ and *γ*_*urban*_ are parameter estimates from Eq 3.

In addition to forecasting trends in WASH-related mortality and water collection times, we estimate the magnitude of economic losses associated with the lack of access to water and sanitation services over the simulation horizon. Conceptually, the economic benefits of access to water services for households consist of health benefits, time-savings, and aesthetic benefits. Framing the economic consequences associated with improving access to water services as ‘reduced economic losses’ instead of ‘economic gains’ reinforces a donor perspective in considering global challenges. Framing the economic consequences as ‘reduced economic losses’ assumes the baseline is a world in which all households have access to piped water and sanitation services, a state of the world that has never existed [14].

Country-level data on households’ willingness to pay for aesthetic and quality of life benefits associated with improved water services are not available. Our estimates of economic losses include only losses associated with WASH-related mortality, morbidity, and water collection time (Eq 7).

$$Losses_{it}^{WSH}=H_{it}+T_{it}$$ (7)

where ${Losses}_{it}^{WSH}$ is the total WASH-related economic losses in country *i* in period *t* and *H*_*it*_ are *T*_*it*_ are the health- and time-related losses, respectively, associated with not having access to adequate water and sanitation services.

The economic losses associated with WASH-related mortality are estimated using the economic concept of the value of statistical life (VSL). (See [14], Annex 10.1) for a detailed description of how we forecast the VSL used in the simulation model). Specifically, the economic losses associated with WASH-related mortality are calculated by multiplying the number of projected WASH-related deaths each year in each country by the forecast VSL for that country (Eq 8). Owing to a lack of country-level data on disease incidence rates, we estimate the economic losses associated with WASH-related morbidity as a fraction of the WASH-related mortality losses. Following [15] we assume that morbidity losses are 25 percent of mortality losses and vary this from 10 percent to 40 percent in our sensitivity analysis.

$$H_{it}=Pop_{it}\cdot d_{it}^{WSH}\cdot VSL_{it}\cdot \left(1+f_{morb}\right)$$ (8)

where *H*_*it*_ is the WASH-related mortality and morbidity losses in country *i* in period *t*; *Pop*_*it*_ is the population in country *i* in period *t*; $d_{it}^{WSH}$ is the WASH-related death rate in country *i* in period *t* from Eq 5; *VSL*_*it*_ is average value of statistical life in country *i* in period *t*; and *f*_*morb*_ is the fraction of morbidity burden.

The economic losses associated with the time households spend collecting water are estimated by multiplying the total amount of time households spend collecting water each year by the opportunity cost of time for each country (Eq 9). Following [14], we assume that the opportunity cost of time is a fraction of the average per capita GDP of the bottom 80 percent of the income distribution to account for the fact that lower income households are more likely to spend time collecting water than high income households.

$$T_{it}=\left(\frac{Pop_{it}}{hhsize_{it}}\right)\cdot 2^{trips}\cdot t_{it}^{collection}\cdot v_{it}^{t}$$ (9)

where *T*_*it*_ are the WASH-related time losses in country *i* in period *t*; *Pop*_*it*_ is the population of country *i* in period *t*; *hhsize*_*it*_ is the average household size in country *i* in period *t*; $t_{it}^{collection}$ is the average one-way water collection time from Eq 6; and $v_{it}^{t}$ is the opportunity cost of time. Thus, both the economic value of time-savings and the VSL increase as GDP per capita increases over the forecast period.

In a departure from our previous work, we model the potential impact of changes in temperatures due to climate change on the trends we forecast. In particular, we include changes in temperature in our simulation model in two ways. First, several studies suggest that climate change, and in particular changes in temperature, affect economic growth [16, 17, 18, 19]. We thus combine estimates of the association between changes in temperature and economic growth with projections of temperature under different climate scenarios to forecast the impact of climate change on economic growth. Therefore, the potential impact of climate change on economic growth indirectly affects our forecasts of the underlying trends in coverage, WASH-related mortality, and time households spend collecting (Eqs 4–6) water as well as our estimates of health and non-health related losses (Eqs 7–9). Recent studies have also found a robust association between increases in temperature and increased incidence of diarrhoea and other waterborne diseases [20, 21, 22]. In Eq 5, we also directly include the impact of changes in temperature on WASH-related mortality.

### 3.1 Base case scenario and sensitivity analysis

In the base case the WASH mortality rate and annual WASH-related deaths for each country are forecast over the simulation period using the parameter estimates obtained in Eqs 1 and 2 and the assumption that countries’ GDP increase at the same average rate as they did over the historical period 1950–2008. We adjust these forecasts of GDP for projected changes in temperature using the central estimates of the association between temperature and GDP from [18]. We use the association between WASH-related mortality and changes in temperature from [23].

We then use Monte Carlo analysis to test the sensitivity of the results to the assumptions about GDP growth as well as the strength of the association between GDP, coverage, urbanisation, and WASH-related mortality. In particular, in the Monte Carlo simulation we draw the average annual growth rate for each country from a normal distribution with a mean of 3.7 percent and a standard deviation of 1.3 percent. This reflects the distribution of GDP growth rates in sub-Saharan Africa from 1990 to 2008, a period over which GDP growth among countries in the region varied considerably. We also vary the strength of the association between GDP, coverage, urbanisation, and WASH-related mortality using the 95 percent confidence intervals of the parameter estimates obtained from Eqs 1 and 2. With respect to changes in temperature, we vary the strength of the association between change in temperature and GDP using the upper and lower bound estimates presented in [18]. Our forecasts of temperature span the 10^th^ and 90^th^ percentiles of the IPCC’s A2 climate change storyline and scenario family. Forecasts of temperature under the B1 storyline and scenario family are also examined. We draw 1,000 realisations of the model parameters to obtain a distribution of outcomes from the simulation model. (S4 Appendix lists the parameters used in the Monte Carlo simulations, their upper and lower bounds, and the type of distribution assumed).

### 3.2 Data

The regression analyses (Eqs 1 and 2) use country-level data from different global data sets [5]. WASH-related mortality (i.e. the ratio of total number of deaths due to WASH to total number of deaths) was calculated for 2002 and 2008 using the methodology from the WHO’s Environmental Burden of Disease (EBD) project. Data for 2004 WASH-related mortality were taken directly from the EBD. Data for coverage with improved sources and for piped water services from 1990 to 2015 were obtained from the JMP. Adult literacy and fertility rates were obtained from the United Nations [13], while governance variables were obtained from the Center for Systemic Peace’s Integrated Network for Societal Conflict Research (http://www.systemicpeace.org/inscrdata.html). These governance variables included measures of the extent of democracy and autocracy, years since regime change, and an indicator for a successful coup in the last five years. Income inequality was measured as the percentage of national GDP for the lowest 80 percent of the income distribution, which was obtained from the [24]. The country-level data for water collection time were obtained from the Demographic and Health Survey (DHS), the Multiple Indicator Cluster Survey (MICS), and the World Health Surveys (WHS) of the WHO. For the simulations, we use urbanisation and population projections from the United Nations Population Division [13]. Finally, country-level projections of changes in temperature were obtained for countries in SSA from the World Bank Climate Knowledge Portal (https://climateknowledgeportal.worldbank.org).

## 4. Results

**Fig 3:**
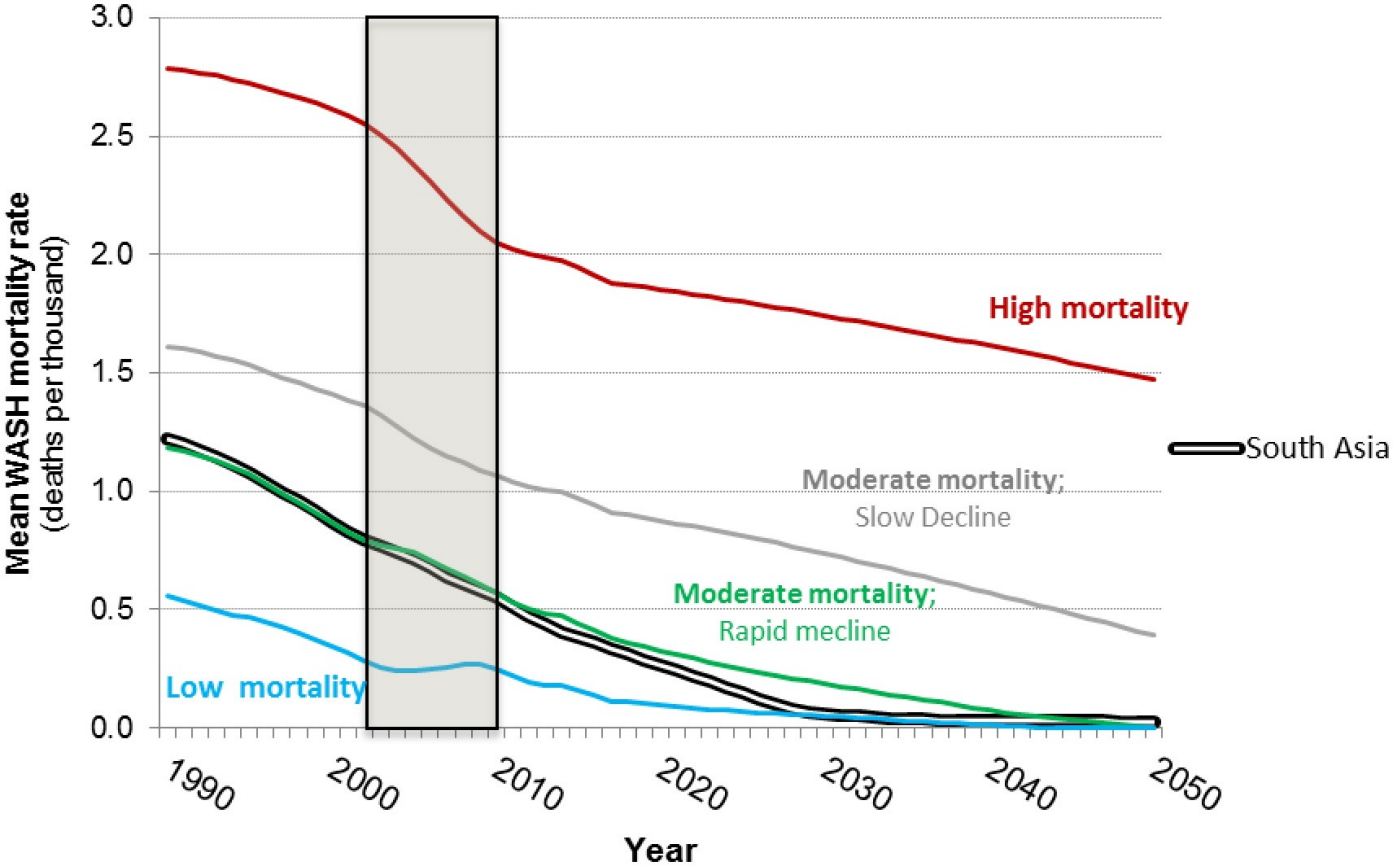
Base case projections of WASH mortality rates for four groups of sub-Saharan Africa countries and South Asia (1990–2050). Shaded area denotes observed data.

**Fig 4:**
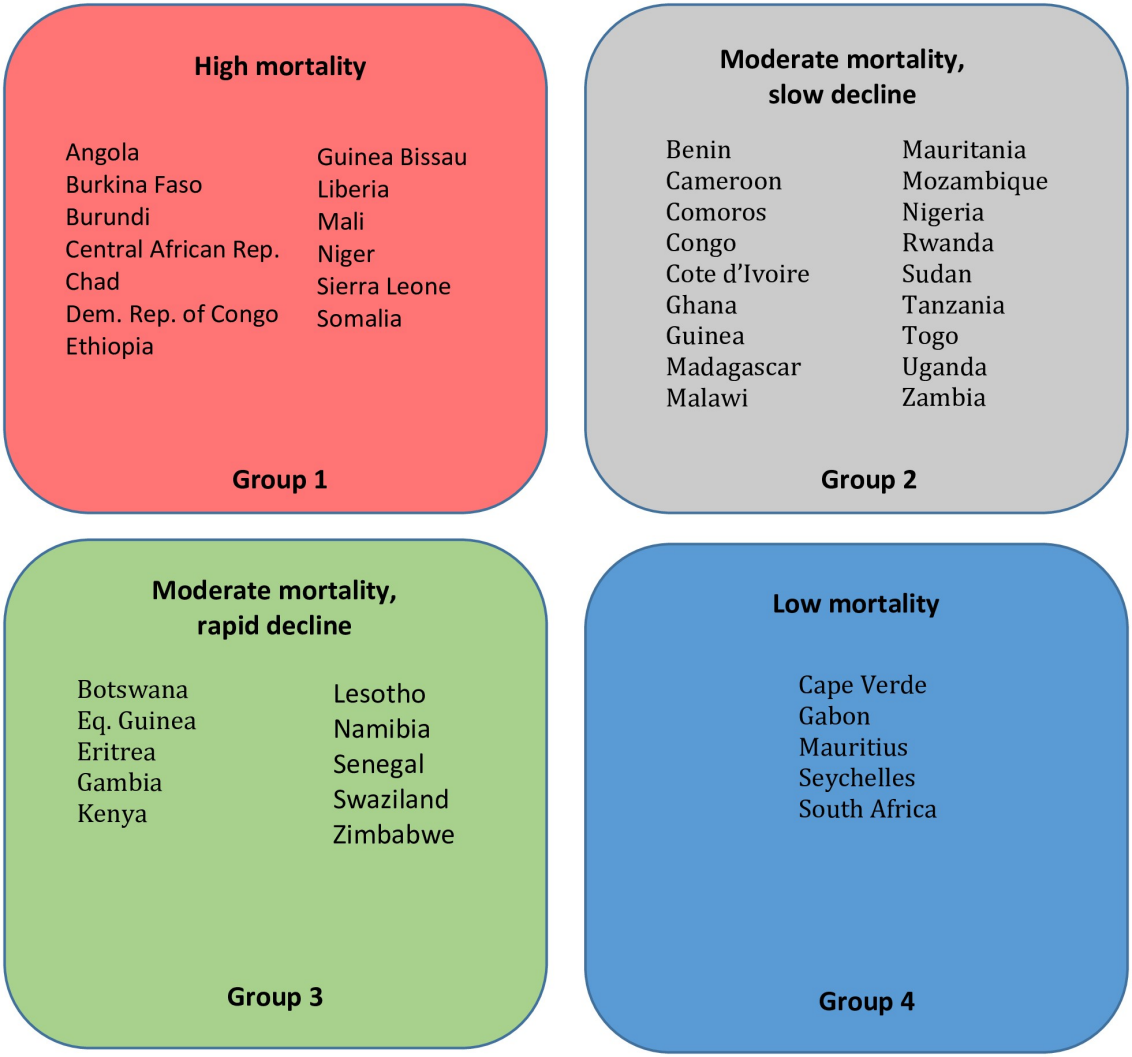
Four groups of countries in sub-Saharan Africa on different WASH mortality trajectories.

Fig 3 shows the base case projections of the population weighted average WASH-related mortality rate for the four groups of sub-Saharan Africa countries. Countries in the first group currently have high WASH-related mortality rates (approximately 2 deaths per 1,000 annually), and the projections indicate that these rates will decline by about 25 percent by 2050. In the base case the projections show that the annual WASH mortality rate will be approximately 1.5 deaths per thousand annually in 2050. This is substantially higher than the WASH mortality rate in the other three groups of sub-Saharan Africa countries today. Countries in this first group include Angola, Burkina Faso, Burundi, Central African Republic, Chad, DRC, Ethiopia, Guinea Bissau, Liberia, Mali, Niger, Sierra Leone, and Somalia (Fig 4).

Countries in the second group currently have moderate WASH mortality rates (>1 death per thousand), and these rates decline steadily (by over 50 percent) over the simulation period to approximately 0.5 deaths per 1,000. Many countries in sub-Saharan Africa fall into this second group, including Benin, Cameroon, Comoros, Congo, Cote d’Ivoire, Ghana, Guinea, Madagascar, Malawi, Mauritania, Mozambique, Nigeria, Rwanda, Sudan, Tanzania, Togo, Uganda, Zambia, and Zimbabwe (Fig 4).

Countries in the third group start with moderate mortality rates (0.5 deaths per thousand), just above the WASH mortality in South Asia today. These rates decline significantly over the simulation period, reaching negligible levels by 2050. Countries in this third group include Botswana, Equatorial Guinea, Eritrea, Gambia, Kenya, Lesotho, Namibia, Senegal, Swaziland, and Zimbabwe (Fig 4).

Countries in the fourth group start with relatively low WASH-related mortality (under 0.5 deaths per thousand). Annual WASH-related mortality rates in this group of countries increase between 2002 and 2008 and then are forecast to decline to negligible levels by 2035. With the exception of South Africa, these countries are mostly small (Cape Verde, Gabon, Mauritius, Seychelles, and South Africa) (Fig 4).

**Fig 5:**
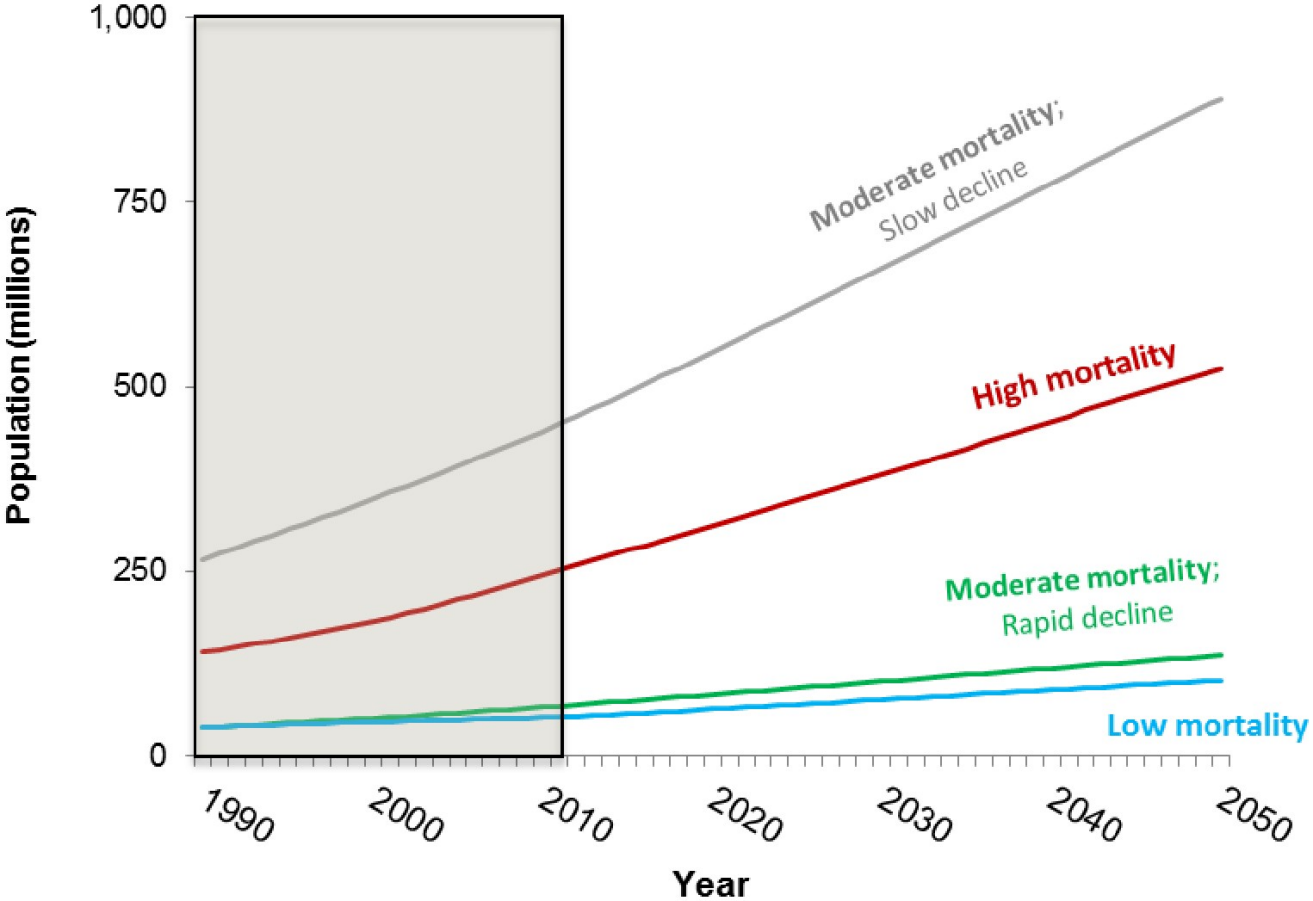
Baseline population forecasts for four groups of sub-Saharan Africa countries. Shaded area denotes observed data.

Fig 5 shows forecasts of the total population (from the United Nations Population Division) of each of the four groups of countries to 2050. Throughout the period, the population of countries in Group 2 is larger than the population of all three other groups combined. By 2050, the combined population of Group 1 (high mortality) and Group 2 (moderate mortality, slow decline) is over 1 billion people.

**Fig 6:**
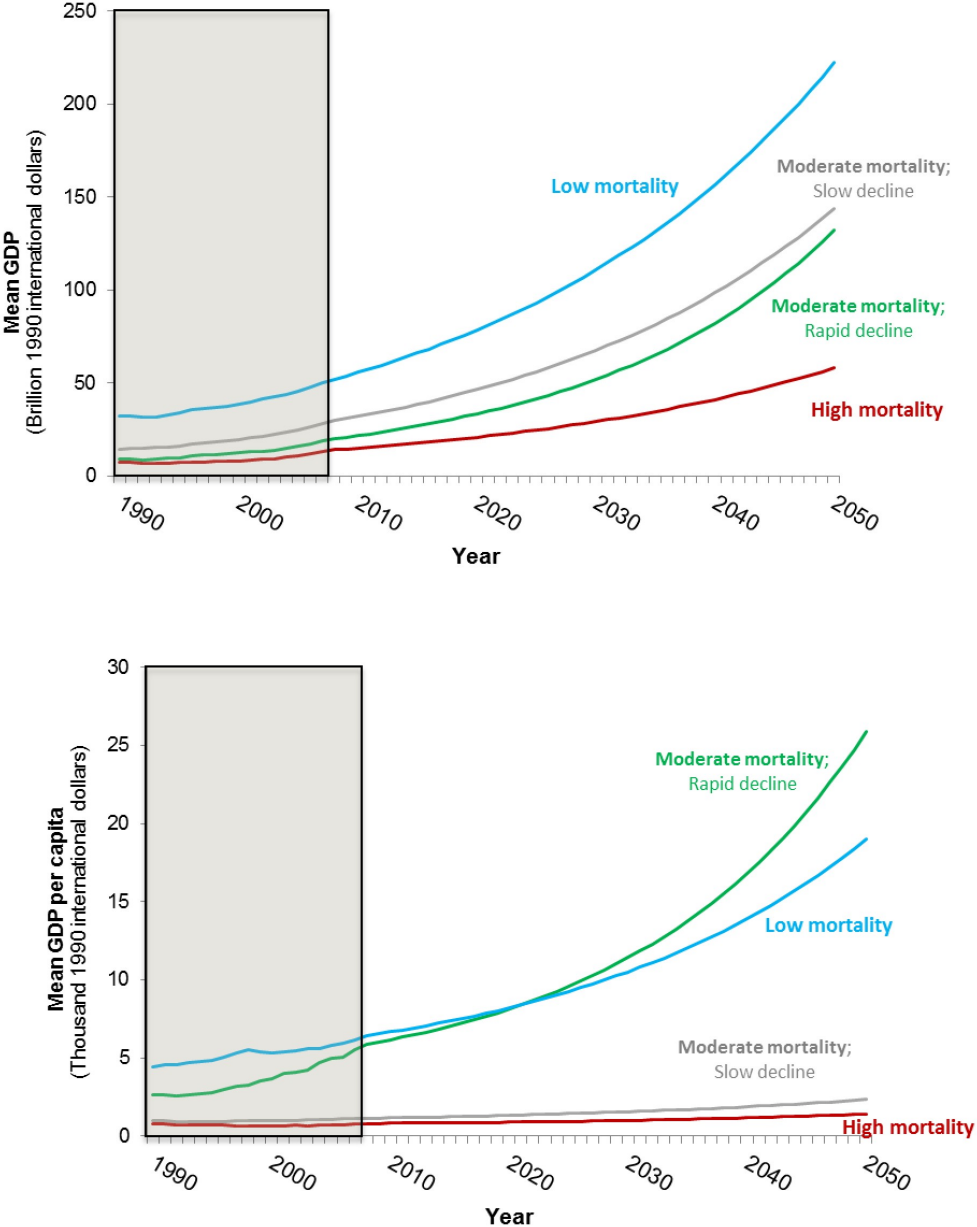
Baseline GDP forecasts for the four groups of sub-Saharan Africa countries (top panel: GDP; bottom panel: GDP per capita).

Fig 6 shows the GDP forecasts based on maintaining historical long-term growth rates for each of the four groups of countries to 2050. GDP per capita in Groups 1 and 2 is lower than in Groups 3 and 4 in 1990. Over the period of the forecast, these groups fall further and further behind Groups 3 and 4 in relative terms. For Groups 1 and 2 the high population forecasts, low initial GDP per capita, and assumed modest economic growth rates combine to hold back improvements in improved water coverage and WASH-related mortality rates.

**Fig 7:**
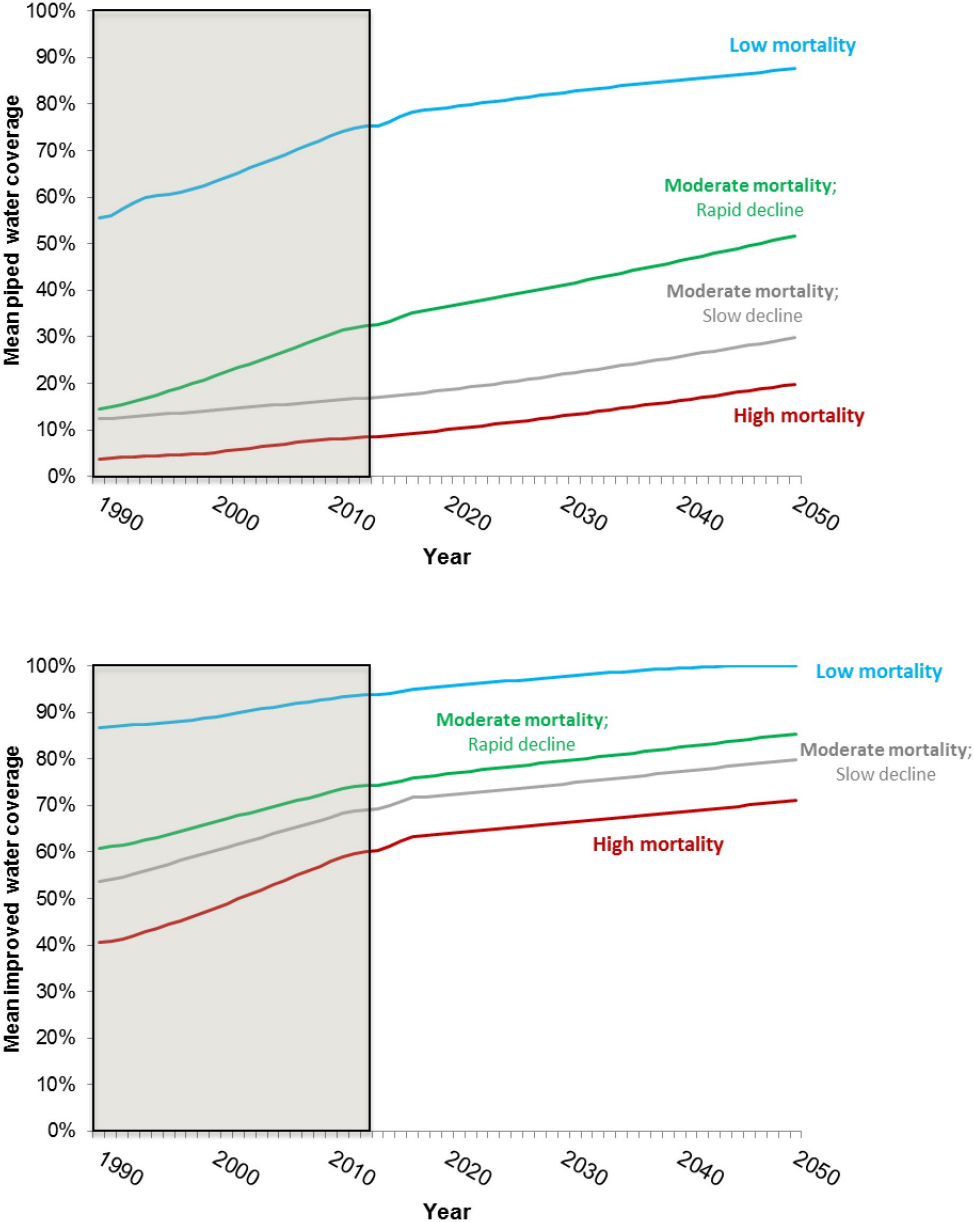
Forecast for piped water coverage for the four groups of sub-Saharan Africa countries (top panel: Piped water coverage; bottom panel: Improved water coverage).

Fig 7 shows the forecasts of piped and improved water coverage by country group to 2050, respectively. Group 4 exhibits much higher levels of both improved water coverage and piped water coverage over the simulation period than the other three groups. Group 2 and Group 3 exhibit similar levels of coverage with improved water sources. However, Group 3 is forecast to have much higher levels of piped water coverage throughout the simulation period. Despite this, we project that less than 50 percent of the population in Group 3 countries will have access to piped water sources in 2050. According to the latest JMP data, approximately half the population in Group 1 has access to an improved water source and only 10 percent of the population in Group 1 has access to a piped water source today. Over the simulation period, we project that coverage levels for both improved and piped water will remain low for Group 1 countries.

### 4.1 Forecasts of WASH-related mortality and water collection times

**Fig 8:**
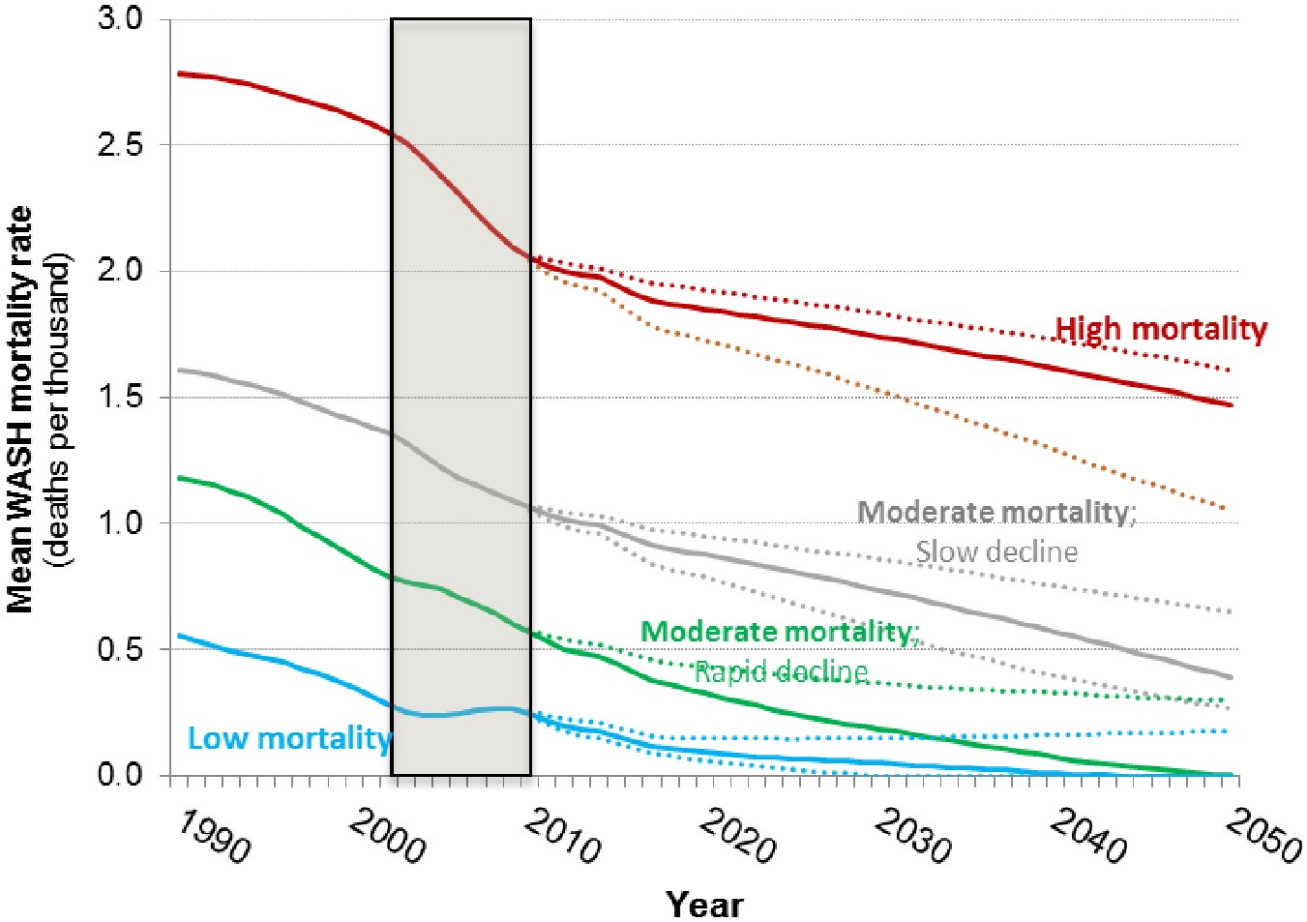
WASH mortality rates for the four groups of sub-Saharan Africa countries; base case projections (solid lines) with the interquartile range from the Monte Carlo simulation (dotted lines).

Fig 8 shows our forecasts for the WASH-related mortality rate in the four sub-Saharan Africa country groups, including the 50 percent confidence interval from our Monte Carlo simulations. The mortality rate for all four groups is trending down, but the baseline mortality rates differ markedly across the four groups. Our forecasts indicate that Group 4 countries, which have low WASH mortality rates today, will see their annual WASH mortality rates decline to negligible levels around 2040, approximately the same WASH mortality rate trajectory as South Asia. The results of our Monte Carlo analysis suggest that the 50 percent confidence interval for the WASH mortality rate in Group 4 countries spans from ‘near zero’ levels as early as 2030 on the optimistic side to a more pessimistic projection of 0.1 deaths per thousand in 2050.

The WASH mortality rate is also forecast to fall to negligible levels over the simulation period in Group 3 countries, but approximately 10 years after South Asia and Group 4 countries. However, the Monte Carlo analysis suggests that the average WASH mortality rate of the countries in Group 3 countries could remain as high as 0.2 deaths per thousand in 2050.

The WASH mortality rate in Groups 1 and 2 is projected to be much higher than the WASH mortality rate in South Asia over the entire simulation period. The Monte Carlo analysis suggests that even on the optimistic side of the interquartile range, the WASH mortality rate in Group 1 still will be well above 1 death per thousand by the end of the simulation period (Fig 8). The WASH mortality rate in Group 2 countries is not projected to reach the current WASH mortality rate in South Asia (approximately 0.5 deaths per thousand) until 2045. The Monte Carlo results suggest the WASH mortality rate in Group 2 could remain as high as 0.6 deaths per thousand at the end of the simulation period.

Our forecasts of WASH-related mortality are most sensitive to assumptions about the elasticity of WASH-related mortality with respect to improved water supply. In particular, the strength of the association between access to improved water and WASH-related mortality explained 60% of the variation in WASH-related mortality in the Monte Carlo simulations. Together, the strength of the associations between access to improved water and WASH-related mortality, urbanization and access to improved water, access to piped water and WASH-related mortality, urbanization and access to piped water, and GDP and access to improved water explained 85% of the variation in the Monte Carlo simulations. In contrast, the elasticities of GDP and WASH-related mortality with respect to temperature explained less 1% of the variation in the Monte Carlo simulations.

**Fig 9:**
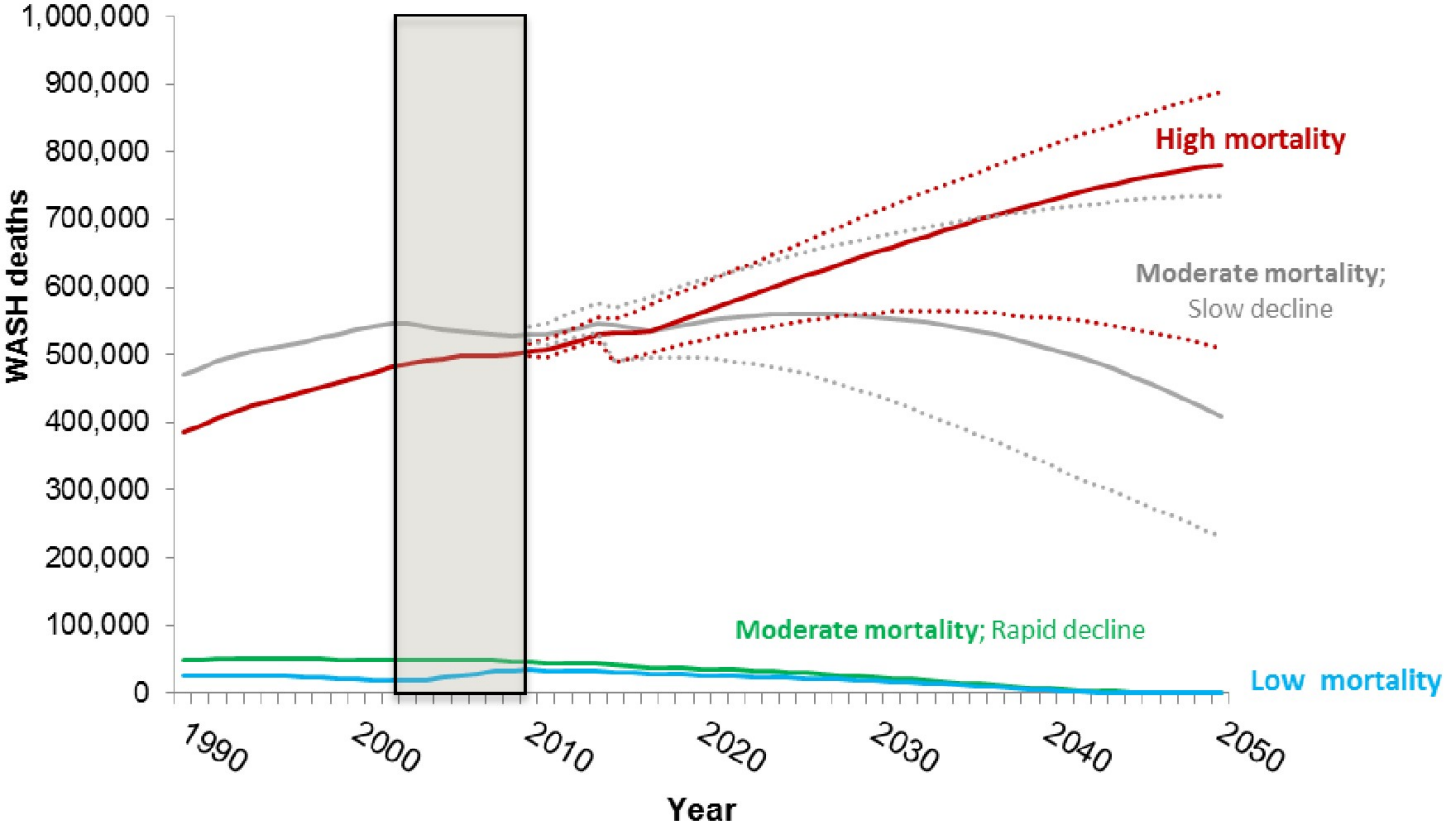
Forecasts of WASH-related deaths for the four groups of sub-Saharan Africa countries; base case projections (solid lines) with the interquartile range from the Monte Carlo simulation (dotted lines). Shaded area denotes observed data. Confidence intervals not shown for low and moderate rapid decline groups because of scale.

Fig 9 presents forecasts of the total annual WASH-related deaths by country group. As shown, total annual deaths in Group 1 are projected to rise continuously over the period. Only on the optimistic side of the interquartile range of our Monte Carlo simulations do WASH-related deaths in Group 1 begin to decline by 2050. In the base case the simulations indicate that the total number of WASH-related deaths in Group 2 countries will peak around 2025 and then decline over the remainder of the simulation period. Total annual deaths in Group 3 and 4 countries are low and decline continuously over the forecast period.

**Fig 10:**
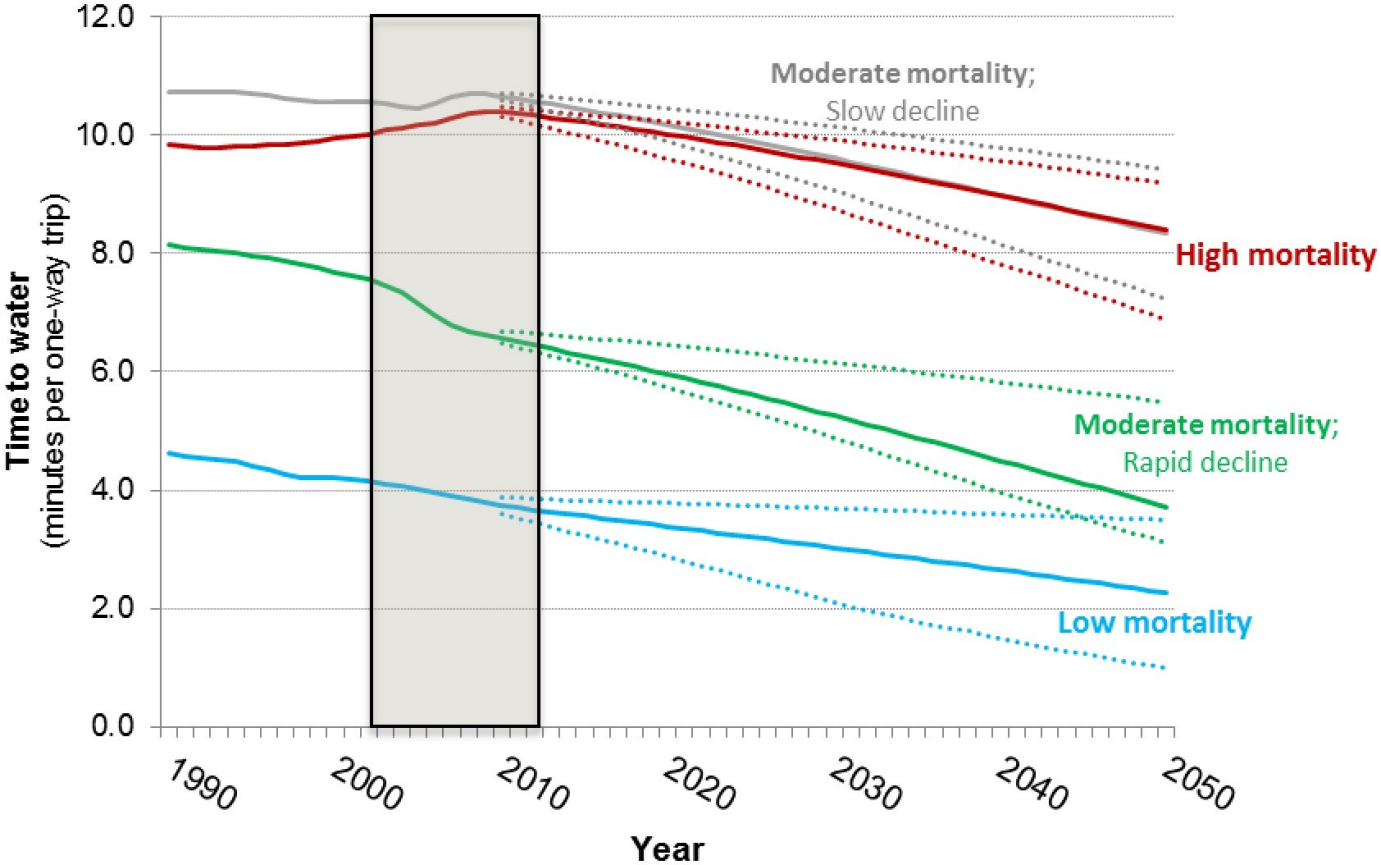
Forecasts of water collection time in four groups of sub-Saharan Africa countries; base case projections (solid lines) with the interquartile range from the Monte Carlo simulation (dotted lines). Shaded area denotes observed data.

Fig 10 shows forecasts of the average (population-weighted) time households spend collecting water in the four country groups. Water collection times are approximately the same in Group 1 and Group 2 countries. While we project that countries in Group 3 and 4 will eliminate WASH mortality by 2050, the forecasts suggest that average water collection times will remain significant in these countries, even on the optimistic side of the interquartile range from our Monte Carlo simulations. This reflects the fact that Group 4 countries are not forecast to achieve universal coverage with piped water by 2050 and that piped water coverage in Group 3 countries remains below 50 percent throughout the simulation period.

### 4.2 Forecasts of economic losses

**Fig 11:**
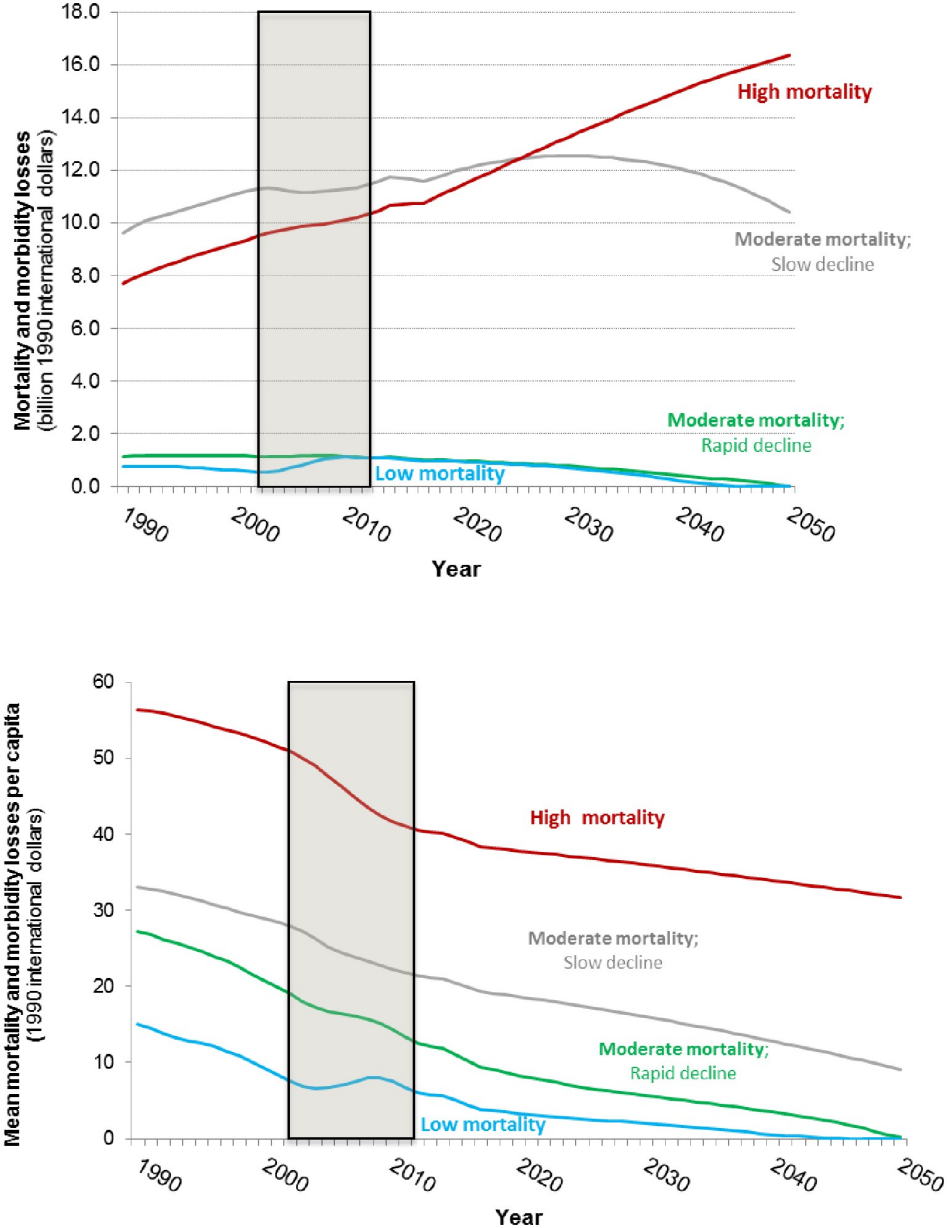
Forecasts of the health-related WASH losses in four groups of sub-Saharan Africa countries (top: Total health losses; bottom: Average per capita health losses). Shaded area denotes observed data.

We next examine the economic consequences of the trends forecast above. Given the uncertainty in the projections, we focus on the relative dynamics of the health- and time-related losses among countries rather than the absolute magnitude of the economic losses. Fig 11 presents forecasts of economic losses associated with WASH mortality and morbidity. Group 3 and 4 countries currently experience low levels of health-related losses and, consistent with the forecasts of WASH-related deaths, we forecast that these countries will eliminate health-related economic losses associated with inadequate access to water services by 2050.

The forecasts suggest that the economic losses associated with WASH-related mortality and morbidity in Group 2 countries will peak in 2030, five years after the peak in WASH-related deaths in this group of countries. This reflects the fact that per capita incomes continue to rise in these countries, increasing the economic value of mortality risk reduction, even as the absolute number of WASH-related deaths and cases of illness decline. The losses associated with WASH mortality and morbidity in Group 1 countries are currently lower than the losses in Group 2 countries, but we forecast that the total economic losses will continue to rise in Group 1 countries over the simulation period, surpassing the losses in Group 2 countries around 2025. Although the total health-related losses in Group 2 countries are forecast to rise over the simulation period, the average per capita health-related losses in these countries is forecast to decrease.

**Fig 12:**
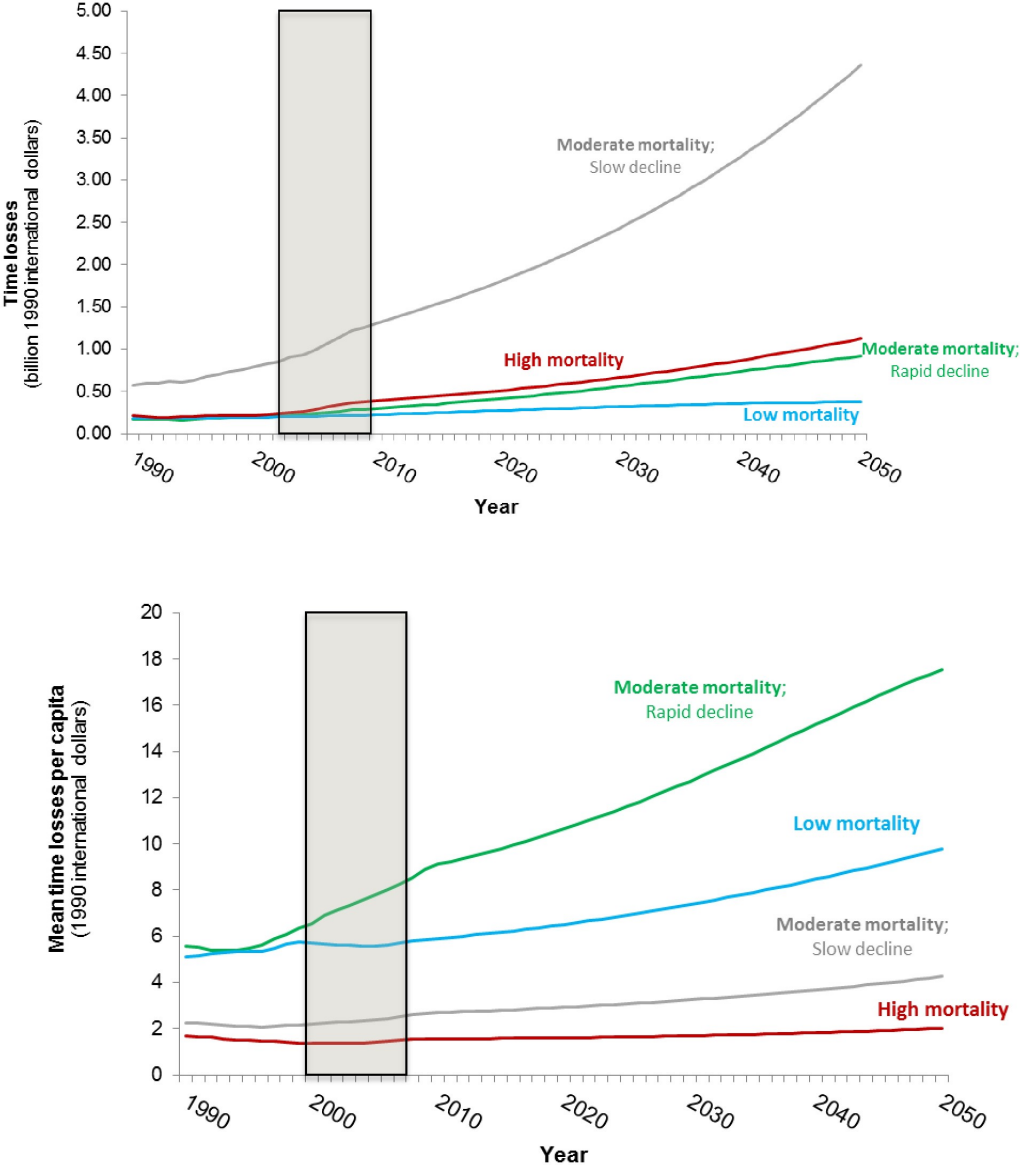
Forecasts of the time-related WASH losses in the four groups of sub-Saharan Africa countries (top: Total time losses; bottom: Average per capita time losses). Shaded area denotes observed data.

Fig 12 shows the forecasts of the economic losses associated with the time households spend collecting water. Despite the fact that Group 3 and Group 4 countries are projected to eliminate health-related losses by 2050, the forecasts suggest that the economic losses associated with water collection time in these countries will rise over the simulation period. This reflects the fact that average water collection times in these countries are forecast to remain positive throughout the simulation period and that the opportunity cost of time increases in these countries as incomes rise.

Time-related losses in Group 1 increase at a similar rate as the time losses in Group 3 countries, but are slightly higher throughout the simulation period. The economic losses associated with the time households spend collecting water are highest in Group 2 countries (Fig 11). Countries in Group 2 are projected to have substantial population growth and had high average collection during the observed data periods, which are not forecast to drop sharply during the simulation period.

Forecasts of average per capita time-related losses tell a more nuanced story (Fig 12, bottom panel). Per capita time losses are lowest in Group 1 countries despite the fact that they exhibit high average water collection times. This reflects both the low opportunity cost of time in these countries as well as the large population in this group. Similarly, despite the fact that the total time losses in Group 2 countries increase substantially over the simulation period, average per capita time losses among countries in this group are forecast to remain relatively stable throughout the simulation period. Per capita time losses are the highest in Group 3 countries, which are projected to have modest population growth over the simulation period and substantial increases in per capita GDP.

**Fig 13:**
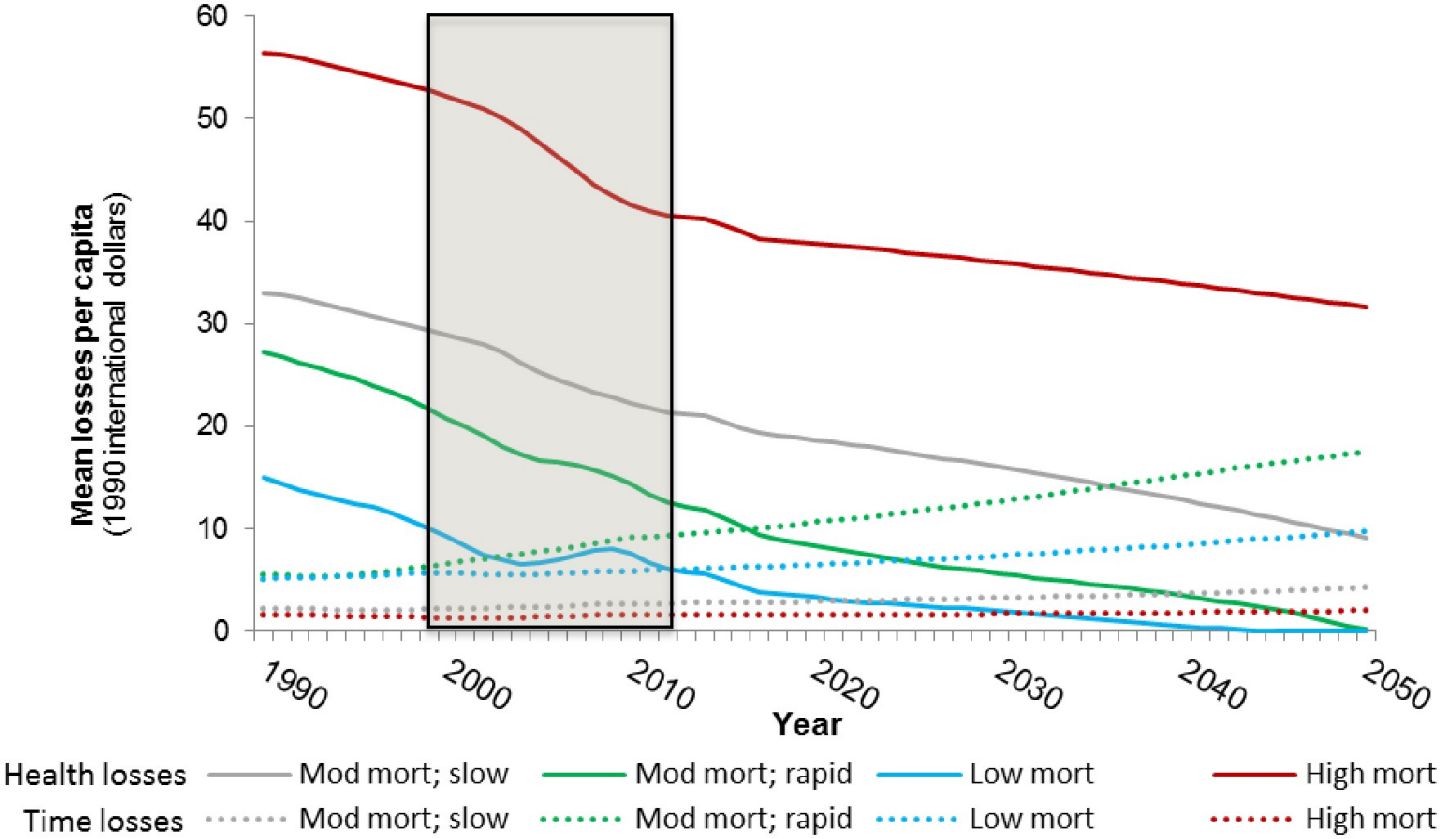
Forecasts of per capita health and time-related WASH losses in the four groups of sub-Saharan Africa countries. Shaded area denotes observed data.

Fig 13 compares the average per capita time- and health-related losses. Per capita health losses are currently much larger than per capita time losses in Group 1 and 2 countries, and the forecasts suggest that health losses will continue to be larger than time losses over the simulation period. This is not surprising given the substantial WASH mortality burden we forecast in these countries over the simulation period. In Group 3 and 4 countries we forecast a different trend. In Group 4 countries, per capita time losses recently surpassed health losses as the primary driver of economic losses associated with inadequate access to water. The forecasts suggest that soon per capita time losses will also be larger than per capita health losses in Group 3 countries.

**Fig 14:**
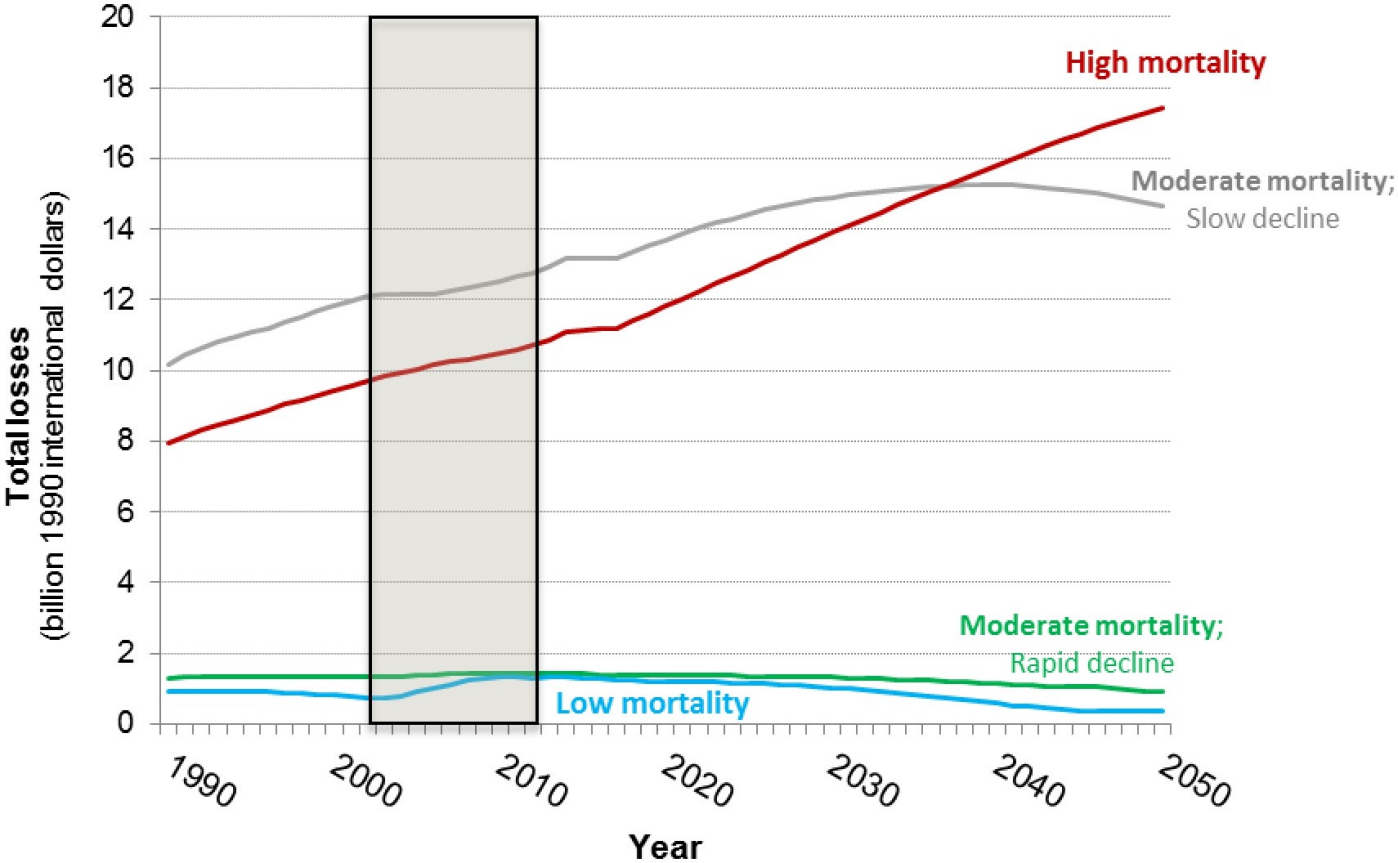
Forecasts of the total WASH-related losses in the four groups of sub-Saharan Africa countries (top: Total losses; bottom: Average per capita losses).

The relative trends observed in the health- and time-related economic losses associated with inadequate access to improved water services are reflected in the forecasts of total economic losses (Fig 14). Driven by large health-related losses, the total losses in Group 1 countries are forecast to increase over the simulation period, surpassing the total economic losses in Group 2 countries by approximately 2035. In the base case, the total losses in Group 2 countries are forecast to peak in 2040, ten years after health-related losses begin to decline in this group of countries. Total losses in Groups 3 and 4 are forecast to remain low, but persist throughout the simulation period despite the fact that these countries largely eliminate WASH-related mortality by 2050. Economic losses in these countries persist because of the lack of universal coverage with piped water services and the increasing opportunity cost of time spent collecting water from outside the home.

## 5. Discussion and conclusions

Our previous findings suggested that over the next four decades sub-Saharan Africa will continue to lag behind other regions in expanding access to improved water sources, reducing WASH-related mortality, and reducing the economic losses associated with lack of access to water and sanitation [5]. This conclusion painted a rather bleak picture for the continent. The results of the simulations presented in this paper, however, tell a more nuanced story about sub-Saharan Africa’s WASH future. Indeed, our forecasts suggest there is considerable heterogeneity in the trajectory of WASH-related mortality and economic losses.

The simulations indicate that the majority of the population in sub-Saharan Africa lives in countries that are making only slow progress in reducing WASH-related mortality, and that the economic losses associated with inadequate access to improved water are likely to remain high well into the future. This includes countries identified as having high WASH-related mortality today and in the future (Group 1) as well as countries with moderate WASH-related mortality today that show only slow progress over the simulation period (Group 2). Even by 2050, WASH-related mortality in these countries is projected to remain above the level of WASH-related mortality in South Asia today. Although the economic losses associated with time spent collecting water outside the home increase in these countries over the simulation period, losses associated with WASH-related mortality and morbidity will continue to drive the total economic losses in these countries over the next three decades.

However, we forecast that some countries in sub-Saharan Africa will reduce the problem of WASH-related mortality and the economic losses associated with inadequate access to improved water supplies to very low levels by 2050. These are countries with low WASH mortality rates today, which decline to near zero levels before the end of the simulation period (Group 4) as well as countries that currently have moderate WASH mortality rates, but are projected to make rapid progress in reducing WASH-related mortality by 2050 (Group 3). Unfortunately, this good news comes from a limited number of countries that are home to only a small fraction of sub-Saharan Africa’s total population. Additionally, while these countries will largely eliminate the health losses associated with inadequate access to improved water sources, economic losses associated with the time households spend collecting water will persist, and indeed will increase, until countries achieve universal access to piped or other in-home water supplies.

The simulations indicate that gradual changes in temperature associated with climate change will not have a significant effect on WASH-related mortality. This result should be interpreted with caution. The simulation model captures only two channels through which climate change might impact economic growth and WASH-related mortality (i.e., change in temperature) and we examine a timeframe over which changes in temperature are projected to be modest. The effects of climate change on economic growth are likely be to highly non-linear and localized and recent evidence suggests that the economies of sub-Saharan Africa are likely to be hardest hit by climate change [18, 16, 17]. Additionally, the model does not capture the impact of catastrophic events such as floods, cyclones, and droughts that are predicted to occur more frequently with climate change and that may have important effects on economic growth, water security, and households’ access to water, sanitation, and other health-related infrastructure and services [25, 26, 27].

Overall, the results of our simulations have important implications for the planning of WASH interventions in sub-Saharan Africa. Given the trends forecast over the next three decades, how can the international community best target resources to countries that lag behind? Many countries in Group 2 have stable governments and emerging economies. However, countries with the highest WASH mortality rates and stagnant progress (Group 1) tend to have fragile governments and either weak economies or heavy reliance on mineral and oil exports. In the base case projections, countries in Group 1 account for over 65 percent of the total WASH-related deaths in sub-Saharan Africa in 2050.

The simulation results presented in this paper also highlight the importance of non-health benefits (e.g. the burden of time spent collecting water) associated with improved water supply. Even as countries reduce or eliminate the disease burden associated with inadequate access to water and sanitation infrastructure, WASH-related economic losses will persist as rising incomes increase the opportunity cost of time spent collecting water from outside the home. Sustainable Development Goal 6 explicitly recognizes the importance of time saving, focusing on safely managed services, which are defined as services that are “located on premises, available when needed and free from contamination” [28]. Nevertheless, policy-makers will need to pay careful attention to non-health benefits of improved water supply when evaluating investments to improve access to water and sanitation infrastructure. To our knowledge, however, there are few empirical estimates of the opportunity cost of time spent collecting water in sub-Saharan Africa that are needed to facilitate such investment analysis. (Exceptions include [29] and [30]). This is a clear area for future research.

The forecasts also suggest that access to water and sanitation services in sub-Saharan Africa will remain low through 2050 if current trends and background associations remain stable. In particular, our simulations indicate that only Group 4 countries will achieve universal access to improved water sources by 2050 and that none of the groups will achieve universal access to piped water on premises by 2050. Indeed, we forecast access to piped water on premises to be below 50% in all but Group 4 countries in 2050. This highlights the enormity of the challenge of achieving the Sustainable Development Goals’ aspiration of ensuring universal access to safely managed water and services by 2030.

Finally, we emphasise that there is nothing inevitable about the forecasts of WASH-related mortality and economic losses due to poor WASH conditions that are presented in this paper. These forecasts simply indicate the direction in which baseline economic growth and demographic drivers are leading coverage and mortality rates. Policy interventions and government commitment can influence these trajectories. But these forecasts are helpful for understanding the scale of the challenge and for focusing attention on where the challenge is likely to be greatest.

## Supporting information

S1 Appendix Summary of WASH mortality data (deaths per thousand). [12].
(DOCX)

S2 Appendix Differences in WASH-related mortality in sub-Saharan Africa and South Asia. [5, 8, 11, 12, 24, 31–48].
(DOCX)

S3 Appendix Baseline economic growth and population forecasts. [31, 13].
(DOCX)

S4 Appendix Summary of parameters, parameter ranges, and distributional assumptions used in the Monte Carlo simulations. [14].
(DOCX)

S1 Data (XLSX)
