# Supplementary material for: Forecasts of mortality and economic losses from poor water and sanitation in sub-Saharan Africa
Source: PLoS One. 2020 Mar 20;15(3):e0227611. doi: 10.1371/journal.pone.0227611 (PMC7083270; doi:10.1371/journal.pone.0227611)
Supplement: S1 Appendix — [12]. (DOCX) [file pone.0227611.s001.docx]

Appendix 1 Summary of WASH mortality data (deaths per thousand)

| **Country** | **2002^*^** | **2004^**^** | **2008^*^** | **Country** | **2002^*^** | **2004^**^** | **2008^*^** |
| --- | --- | --- | --- | --- | --- | --- | --- |
| Angola | 3.27 | 3.22 | 3.01 | Liberia | 2.51 | 2.11 | 1.31 |
| Benin | 1.63 | 1.58 | 1.20 | Madagascar | 1.55 | 1.57 | 0.76 |
| Botswana | 0.23 | 0.25 | 0.27 | Malawi | 1.91 | 1.92 | 1.25 |
| Burkina Faso | 2.73 | 2.54 | 1.94 | Mali | 2.98 | 2.87 | 2.41 |
| Burundi | 1.92 | 1.89 | 1.84 | Mauritania | 1.21 | 1.13 | 1.06 |
| Cameroon | 1.40 | 1.32 | 1.48 | Mauritius | 0.01 | 0.01 | 0.02 |
| Cape Verde | 0.46 | 0.32 | 0.25 | Mozambique | 2.21 | 1.12 | 1.04 |
| Central African Republic | 1.48 | 1.48 | 1.37 | Namibia | 0.47 | 0.42 | 0.33 |
| Chad | 3.13 | 3.01 | 2.94 | Niger | 3.35 | 3.02 | 2.35 |
| Comoros | 0.86 | 0.65 | 0.89 | Nigeria | 1.69 | 1.60 | 1.41 |
| Congo | 0.63 | 0.52 | 0.76 | Rwanda | 1.51 | 1.44 | 1.32 |
| Cote d'Ivoire | 1.10 | 1.11 | 1.11 | Senegal | 0.77 | 0.72 | 0.64 |
| Dem. Rep. of the Congo | 2.76 | 2.60 | 2.24 | Seychelles | 0.02 | 0.02 | 0.02 |
| Equatorial Guinea | 1.04 | 1.40 | 1.05 | Sierra Leone | 2.42 | 2.15 | 1.62 |
| Eritrea | 0.95 | 0.88 | 0.63 | Somalia | 2.59 | 2.53 | 2.43 |
| Ethiopia | 1.68 | 1.66 | 1.62 | South Africa | 0.35 | 0.47 | 0.71 |
| Gabon | 0.37 | 0.36 | 0.41 | Sudan | 0.63 | 0.75 | 0.94 |
| Gambia | 0.84 | 0.87 | 0.80 | Swaziland | 0.73 | 0.58 | 0.52 |
| Ghana | 1.21 | 1.11 | 1.06 | Tanzania | 1.14 | 1.12 | 1.00 |
| Guinea | 1.47 | 1.41 | 1.27 | Togo | 1.25 | 1.21 | 0.85 |
| Guinea Bissau | 1.97 | 1.98 | 2.00 | Uganda | 1.21 | 1.26 | 1.35 |
| Kenya | 0.92 | 0.89 | 0.81 | Zambia | 1.95 | 1.51 | 1.40 |
| Lesotho | 0.81 | 0.68 | 0.43 | Zimbabwe | 0.78 | 0.78 | 0.49 |
| Liberia | 2.51 | 2.11 | 1.31 |  |  |  |  |

^*^ Calculated using the methodology from the WHO’s Environmental Burden of Disease project.

^**^ Obtained directly from [12].
