# Supplementary material for: Forecasts of mortality and economic losses from poor water and sanitation in sub-Saharan Africa
Source: PLoS One. 2020 Mar 20;15(3):e0227611. doi: 10.1371/journal.pone.0227611 (PMC7083270; doi:10.1371/journal.pone.0227611)
Supplement: S2 Appendix — [5, 8, 11, 12, 24, 31–48]. (DOCX) [file pone.0227611.s002.docx]

Appendix 2 Differences in WASH-related mortality in sub-Saharan Africa and South Asia

It is a puzzle why WASH-related mortality rates are estimated to be so much higher in sub-Saharan Africa than in South Asia. Both sub-Saharan Africa and South Asia have poor WASH conditions, high childhood malnutrition, and high levels of poverty. Moreover, the incidence of several infectious diseases (e.g. pneumonia and diarrhoeal diseases) is similar in sub-Saharan Africa and South Asia. But while sub-Saharan Africa had 23 percent of the global population of children under five years old in 2010, it had about 48 percent of estimated worldwide child deaths [31, 8]. Pneumonia incidence for children under five is comparable in both regions, but mortality rates in sub-Saharan Africa also tended to be two to three times greater than in South Asia [32]. In comparison, South Asia accounted for 27 percent of the worldwide population of children under five years old and had about 33 percent of child deaths [31, 8].

There are many factors that might explain the differences in WASH-related mortality in sub-Saharan Africa and South Asia, including differences in per capita income, healthcare access and quality, access to improved water and sanitation, demographics, fertility, and/or concomitant diseases. Sub-Saharan Africa, excluding South Africa, has the lowest indicators in the world for per capita incomes, headcount poverty, and child mortality [33, 34]. In 2008, nearly 48 percent of the sub-Saharan Africa population lived on less than US$1.25 (PPP, purchasing power parity) per day, compared with 36 percent in South Asia. The proportion of the population living on less than US$2 (PPP) is nearly identical in the two world regions, 69 percent in sub-Saharan Africa compared with 71 percent in South Asia [24]. Yet, differences in income likely are not the full explanation of the differences in WASH-related mortality rates.

Other possible explanations for the divergence in WASH-related mortality rates, and those from other infectious diseases across these regions, include differences in healthcare access and healthcare behaviours. Some protective behaviours include breastfeeding from age 0–2 months, vaccination (e.g. for rotavirus, which causes about 40 percent of diarrhoea hospital admissions among children under five), and adequate vitamin A and zinc [35, 36, 11]. Health-seeking behaviours for the sick may also be low in sub-Saharan Africa compared to South Asia, perhaps partly as a result of the lower population density and lack of healthcare facilities. However, country-level rates of diarrhoea treatment (oral rehydration salts (ORS), homemade ORS remedies, or hospital admissions) do not seem to differ significantly between sub-Saharan Africa and South Asia for countries and years for which data are available [37]. Health-seeking behaviour may also differ between South Asia and sub-Saharan Africa because of social and cultural factors.

There may also be differential impacts in the determinants and magnitude of child mortality across world regions that are due to non-linearities, interactions, and compounding of the types of effects listed above, or because of other health conditions [38, 34]. Other diseases, such as HIV and malaria, are known to exacerbate susceptibility to WASH-related diseases, and the incidence of these conditions is highest in sub-Saharan Africa. On the other hand, rates of childhood malnutrition are much higher in South Asia [34]. Consistent with this, anthropometric shortfall (height for age and weight for age) is almost 70 percent higher in South Asia compared with sub-Saharan Africa [12, 39, 40].

Some recent literature also argues that the striking differences in stunting between children in South Asia and sub-Saharan Africa (the so-called ‘Asian enigma’) can be almost fully explained by the much higher rates of open defecation in the former region, and particularly in India [41, 42]. In fact, over half of people in India practise open defecation, in contrast to much lower rates (~25 percent) across sub-Saharan Africa. The role that diarrhoeal illness plays in malnutrition has long been recognised [43], yet the consensus in the literature has generally been that its contribution is modest, perhaps causing 4 percent of stunting [44].

More recently, however, the public health literature has drawn attention to a condition known as tropical enteropathy, a subclinical disorder of the small intestine that perhaps better explains the connection between sanitation and hygiene and undernutrition [45]. This literature uses evidence from long-term studies to suggest that the primary causal pathway from poor sanitation and hygiene to undernutrition may be through tropical enteropathy rather than diarrhoea [46, 47]. If this is true, then poor sanitation may explain the unexpected differences in stunting in India versus sub-Saharan Africa while explaining little about the mortality gradient from diarrhoea (which might instead relate to factors such as differential prevalence of other deadly infectious diseases, immunisation rates, or health-seeking behaviours discussed above). There may also be a connection between population density and environmental contamination by faeces, and therefore tropical enteropathy.

Finally, the divergence in WASH mortality between sub-Saharan Africa and South Asia may be due to measurement error. The WHO cautions that estimates for different years are not directly comparable because they include: 1) differing vital registration data; 2) different sources of epidemiological data for specific causes; and 3) information on child and adult mortality that varies by year in countries lacking good death registration data. There is also evidence of data discrepancies in the WHO’s burden of disease estimates. For example, the data for India indicate many more deaths from diarrhoea in 2008 relative to 2002 and 2004, but the large increase in reported fatality rates occurs among adults, which seems unlikely [5]. Similarly, countries may differ markedly with respect to the quality of data collection, analysis, and reporting [48].
