# Supplementary material for: Forecasts of mortality and economic losses from poor water and sanitation in sub-Saharan Africa
Source: PLoS One. 2020 Mar 20;15(3):e0227611. doi: 10.1371/journal.pone.0227611 (PMC7083270; doi:10.1371/journal.pone.0227611)
Supplement: S3 Appendix — [31, 13]. (DOCX) [file pone.0227611.s003.docx]

**Appendix 3. Baseline economic growth and population forecasts**

Figure A3-1 shows population projections for sub-Saharan Africa and other regions. While the population in other regions is projected to begin to level off by 2050, the population in sub-Saharan Africa is projected to continue to rise. This increasing trend in population is reflected in the projected population of four large countries in the region: South Africa, Kenya, Nigeria, and the Democratic Republic of the Congo (DRC) (Figure A3-2). Fifty percent of the population of sub-Saharan Africa is projected to live in urban areas by 2030 (Figure A3-3). This level of urbanisation is reached well after Latin America and East Asia, but before South Asia.

Figure A3-4 shows projections of GDP per capita for sub-Saharan Africa and other regions assuming historical growth rates continue. The GDP per capita in both South Asia and sub-Saharan Africa is well below the GDP per capita in Latin America and East Asia. Despite having similar levels of per capita GDP in 1990, South Asia has recently been growing much faster and now has roughly double the per capita GDP of sub-Saharan Africa. This divergence, if it continues, will lead to a roughly threefold difference in GDP per capita by 2050. There is however considerable heterogeneity in the historical economic growth of countries within sub-Saharan Africa. This difference will lead to increasing divergence across countries in the region if it continues into the future (Figure A3-5). For example, South Africa begins in 1990 with somewhat higher per capita GDP than South Asia, but experiences somewhat lower growth rates. Nigeria, Kenya, and the DRC have lower GDP per capita and slower growth rates than South Asia. Per capita GDP will be much higher than it is today in South Africa, Nigeria, and Kenya by 2050 if historical growth rates continue. However, GDP per capita growth in the DRC has been near zero.

South Asia

SSA

Latin America

Figure A3-1. Population estimates from 1980 to 2050 for four regions

*Note: shaded area denotes observed data*

Source: [31].


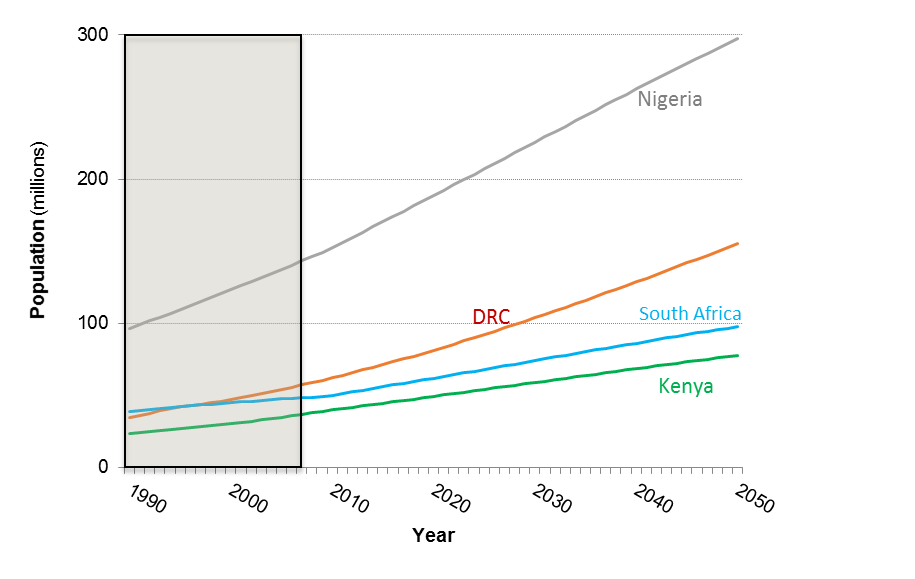


Figure A3-2. Baseline population projections for Nigeria, Democratic Republic of the Congo (DRC), South Africa, and Kenya. Source: [31].


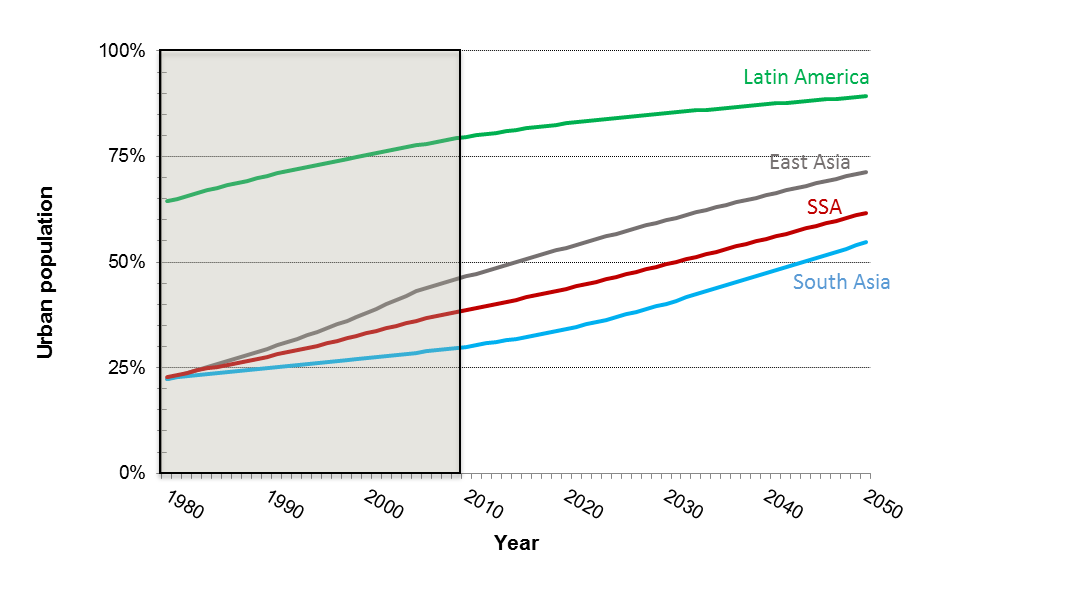
Figure A3-3. Estimates of the percentage of the population living in urban areas from 1980 to 2050 for four regions. Source: [13].


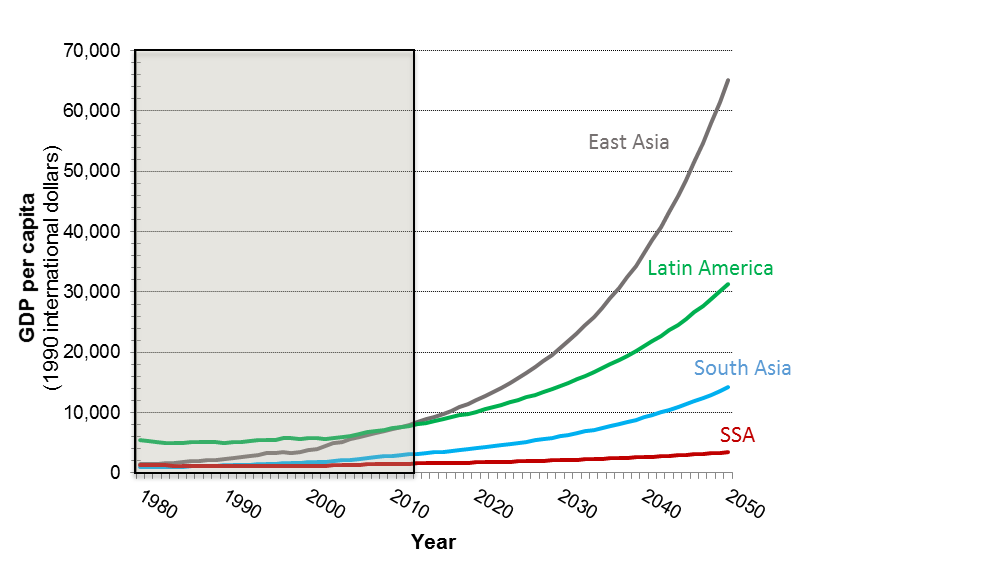


Figure A3-4. Baseline projections of GDP per capita in four regions


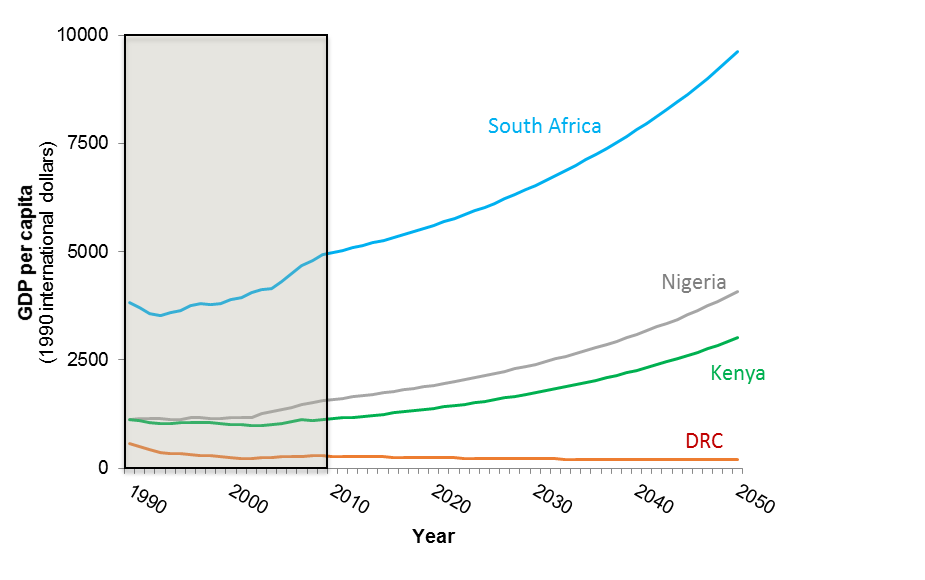


Figure A3-5. Baseline projections of GDP per capita in South Africa, Nigeria, Kenya, and DRC
