# Supplementary material for: Forecasts of mortality and economic losses from poor water and sanitation in sub-Saharan Africa
Source: PLoS One. 2020 Mar 20;15(3):e0227611. doi: 10.1371/journal.pone.0227611 (PMC7083270; doi:10.1371/journal.pone.0227611)
Supplement: S4 Appendix — [14]. (DOCX) [file pone.0227611.s004.docx]

Appendix 4. Summary of parameters, parameter ranges, and distributional assumptions used in the Monte Carlo simulations

|  |  |  | | |  |
| --- | --- | --- | --- | --- | --- |
| **Simulation parameter** | **Base case** | **Lower bound** | **Upper bound** | | **Distribution** |
| **GDP** |  |  |  | |  |
| Annual growth rate | Historical growth^a^ | N/A | N/A | | Normal dist. N(3.6, 1.3) |
| GDP temperature elasticity^b^ | -5% | -20% | 0 | | Uniform dist. |
| **Coverage** |  |  | |  |  |
| Piped water GDP elasticity | 9.5 | 4.5 | | 14.5 | Uniform dist. |
| Piped water % Urban elasticity | 0.35 | 0.1 | | 0.6 | Uniform dist. |
| Improved water GDP elasticity | 4.5 | 0 | | 9 | Uniform dist. |
| Improved water % Urban elasticity | 0.3 | 0.1 | | 0.5 | Uniform dist. |
| **WASH mortality** |  |  | |  |  |
| GDP elasticity | -0.2 | -0.1 | | -0.3 | Uniform dist. |
| Piped water elasticity | -0.02 | -0.01 | | -0.03 | Uniform dist. |
| Improved water elasticity | -0.0275 | -0.01 | | -0.045 | Uniform dist. |
| Temperature elasticity | 0.575 | 0.4 | | 0.75 | Uniform dist. |
| **Time to water** |  |  | |  |  |
| Time to water GDP elasticity | -1.2 | -0.3 | | -2.1 | Uniform dist. |
| Time to water % urban elasticity | -0.06 | -0.02 | | -0.1 | Uniform dist. |
| Average household size | 5 | 4 | | 6 | Uniform dist. |
| Number of trips per day | 1.5 | 1 | | 2 | Uniform dist. |
| **Economic losses** |  |  | |  |  |
| *Health losses* |  |  | |  |  |
| VSL | (*see [14]: Annex 10.1*) | | | | |
| Morbidity as % of mortality (*f_morb_*) | 0.25 | 0.4 | | 0.1 | Uniform dist. |
| *Time losses* |  |  | |  |  |
| Hours per work week | 40 | 30 | | 50 | Uniform dist. |
| Shadow value of labour (fraction of per capita GDP^c^) | 0.25 | 0.1 | | 0.5 | Uniform dist. |
|  | |  | |  |  |

^a^ Average annual growth rate from 1950 to 2008; ^b^ change in GDP growth per 1C increase in temperature; ^c^ see [14] for details.
